# Antibody-independent protection against heterologous SARS-CoV-2 challenge conferred by prior infection or vaccination

**DOI:** 10.1038/s41590-024-01787-z

**Published:** 2024-03-14

**Authors:** Valeria Fumagalli, Micol Ravà, Davide Marotta, Pietro Di Lucia, Elisa B. Bono, Leonardo Giustini, Federica De Leo, Maura Casalgrandi, Emanuele Monteleone, Violette Mouro, Chiara Malpighi, Chiara Perucchini, Marta Grillo, Sara De Palma, Lorena Donnici, Silvia Marchese, Matteo Conti, Hiromi Muramatsu, Stanley Perlman, Norbert Pardi, Mirela Kuka, Raffaele De Francesco, Marco E. Bianchi, Luca G. Guidotti, Matteo Iannacone

**Affiliations:** 1 Division of Immunology, Transplantation, and Infectious Diseases, IRCCS San Raffaele Scientific Institute, Milan, Italy; 2 Vita-Salute San Raffaele University, Milan, Italy; 3 Division of Genetics and Cell Biology, IRCCS San Raffaele Scientific Institute, Milan, Italy; 4 HMGBiotech, Milan, Italy; 5 Charles River Laboratories, Calco, Italy; 6 Istituto Nazionale di Genetica Molecolare (INGM) ‘Romeo ed Enrica Invernizzi’, Milan, Italy; 7 Department of Pharmacological and Biomolecular Sciences, University of Milan, Milan, Italy; 8 Department of Microbiology, Perelman School of Medicine, University of Pennsylvania, Philadelphia, PA USA; 9 Department of Microbiology and Immunology, University of Iowa, Iowa City, IA USA; 10 Department of Pediatrics, University of Iowa, Iowa City, IA USA; 11 Experimental Imaging Centre, IRCCS San Raffaele Scientific Institute, Milan, Italy

**Keywords:** Immunological memory, RNA vaccines, Viral infection, Viral infection

## Abstract

Vaccines have reduced severe acute respiratory syndrome coronavirus 2 (SARS-CoV-2) morbidity and mortality, yet emerging variants challenge their effectiveness. The prevailing approach to updating vaccines targets the antibody response, operating under the presumption that it is the primary defense mechanism following vaccination or infection. This perspective, however, can overlook the role of T cells, particularly when antibody levels are low or absent. Here we show, through studies in mouse models lacking antibodies but maintaining functional B cells and lymphoid organs, that immunity conferred by prior infection or mRNA vaccination can protect against SARS-CoV-2 challenge independently of antibodies. Our findings, using three distinct models inclusive of a novel human/mouse ACE2 hybrid, highlight that CD8^+^ T cells are essential for combating severe infections, whereas CD4^+^ T cells contribute to managing milder cases, with interferon-γ having an important function in this antibody-independent defense. These findings highlight the importance of T cell responses in vaccine development, urging a broader perspective on protective immunity beyond just antibodies.

## Abstract (web summary)

Here the authors use three different mouse models to show that prior infection or mRNA vaccination can protect against severe acute respiratory syndrome coronavirus 2 (SARS-CoV-2) independently of antibodies, highlighting the importance of T cell-derived interferon-γ (IFN-γ) in host defense and the need to consider this measure of protection in vaccination.

## Main

Neutralizing antibody responses have been viewed traditionally as the main bulwark against severe acute respiratory syndrome coronavirus 2 (SARS-CoV-2) re-infection for vaccinated or previously infected individuals^1–3^. While the wider effector functions of antibodies extend beyond neutralization^4,5^, there is an inherent vulnerability owing to the transient nature of these neutralizing antibodies^6–8^. This vulnerability is further exacerbated by the emergence of viral variants that can evade these responses^9–11^. This has led to a prevailing notion that vaccines might necessitate periodic updates to counteract the evolving variants of concern (VOCs) and might require consistent boosting to maintain efficacy.

Conversely, T cells are acknowledged for their role in averting severe manifestations of COVID-19, and their contribution to immunity has been echoed across multiple animal studies^12,13^. The lingering question, however, revolves around the capability of T cells to provide robust protection against a heterologous SARS-CoV-2 challenge in the absence of antibodies. This scenario is not only hypothetical; it could manifest in cases where VOCs dodge neutralizing antibodies, or in patients like those with agammaglobulinemias^14–16^, or those undergoing treatments for cancer or multiple sclerosis who are treated with B cell-depleting agents^17–20^.

Evaluating the protective role of T cells independent of antibodies is fraught with challenges. For example, mice inherently deficient in B cells might not only be devoid of antibodies but may also have anomalies in their lymphoid tissue architecture^21–23^, altered macrophage phenotype^24,25^ and defective T cell responses^26^. Additionally, using adoptive transfer of T cells as a methodology poses its own set of challenges, potentially failing to station adequate numbers of T cells where and when they are most needed.

To overcome these limitations, we took advantage of D_H_LMP2a mice, which possess B cells and retain normal lymphoid tissue architecture, yet are devoid of both surface and circulating immunoglobulins^27^. Using three independent mouse models, including one hybrid (hy) angiotensin-converting enzyme 2 (ACE2) knock-in mouse model generated ad hoc for this study, we show here that prior infection or mRNA vaccination can indeed offer protection against heterologous SARS-CoV-2 challenge, all while circumventing the need for antibodies.

## Results

### Antibody-independent protection in K18-hACE2 transgenic mice

**Extended Data Fig. 1:**
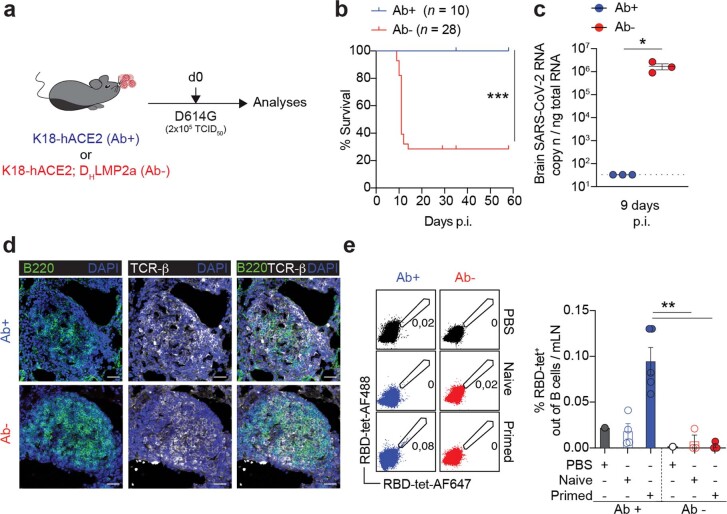
Antibody-independent protection against heterologous SARS-CoV-2 challenge conferred by prior infection in K18-hACE2 transgenic mice. (**a**) Experimental setup. Antibody-sufficient (Ab^+^, *n* = 3-10) and antibody-deficient (Ab^−^, *n* = 3–28) K18-hACE2 transgenic mice were infected with a target dose of 2 × 10^5^ TCID_50_ of SARS-CoV-2 D614G through aerosol exposure. Brain was collected and analyzed 9 days postchallenge. (**b**) Survival curve of Ab^+^ (*n* = 10, blue line) and Ab^−^ mice upon infection (*n* = 28, red line). **(c)** Quantification of SARS-CoV-2 RNA in the brain of the indicated mice. RNA values are expressed as copy number per ng of total RNA and the limit of detection is indicated as a dotted line. *n* = 3. (**d, e**) Experimental setup as described in Fig. 1. (**d**) Representative confocal immunofluorescence staining of lung sections from Ab^+^ (upper panels) and Ab^−^ (lower panels) mice 4 days after re-challenge. Cell nuclei are depicted in blue, B220^+^ cells in green and TCR-β^+^ cells in white. Scale bar, 30 μm. (**e**) Representative flow cytometry plots (left) and frequency (right) of RBD-specific B cells in the mLN of indicated mice 4 days post re-challenge (pre-gated on live^+^/CD4^−^/ CD8^−^/ B220^+^/CD19^+^ cells). *n* as indicated in Fig. 1a. Data are expressed as mean ± SEM. Data are representative of at least 2 independent experiments. *p value < 0.05, **p value < 0.01, ***p value < 0.001; log-rank (Mantel-Cox) test (**b**); Two-tailed unpaired t-test (**c**); Kruskal-Wallis test followed by uncorrected Dunn’s test, each comparison stands alone (**e**). Source data

To determine if protection against heterologous SARS-CoV-2 challenge exists independently of antibodies, we initially made use of K18-hACE2 transgenic mice^28^. These mice, known for expressing human ACE2 (hACE2) predominantly in epithelial cells under the control of the cytokeratin 18 (KRT18) promoter, were crossbred with D_H_LMP2a mice, which maintain B cells and normal lymphoid tissue architecture yet lack both surface and circulating immunoglobulins^27^. Hereinafter, these are referred to as antibody-deficient (Ab^−^) mice. Using a specialized inhalation tower system^29^, both antibody-sufficient (Ab^+^) and Ab^−^ K18-hACE2 transgenic mice were exposed to aerosolized SARS-CoV-2 (D614G) under consistent pressure, temperature and humidity (Extended Data Fig. 1a). This exposure leads to robust viral replication in the respiratory tract^29^. However, unlike intranasal inoculation, this method averts fatal viral neuroinvasion^29^. Accordingly, all Ab^+^ K18-hACE2 transgenic mice survived SARS-CoV-2 infection without detectable viral RNA in their brains (Extended Data Fig. 1b,c). Conversely, ~70% of Ab^−^ K18-hACE2 transgenic mice died between 9 and 12 days postinfection, revealing pronounced viral RNA in their brains (Extended Data Fig. 1b,c). These results are consistent with the unusually elevated hACE2 expression in K18-hACE2 transgenic mice^28,29^, highlighting the protective role mucosal antibodies have in defending against mucosal infections^30^.

**Fig. 1:**
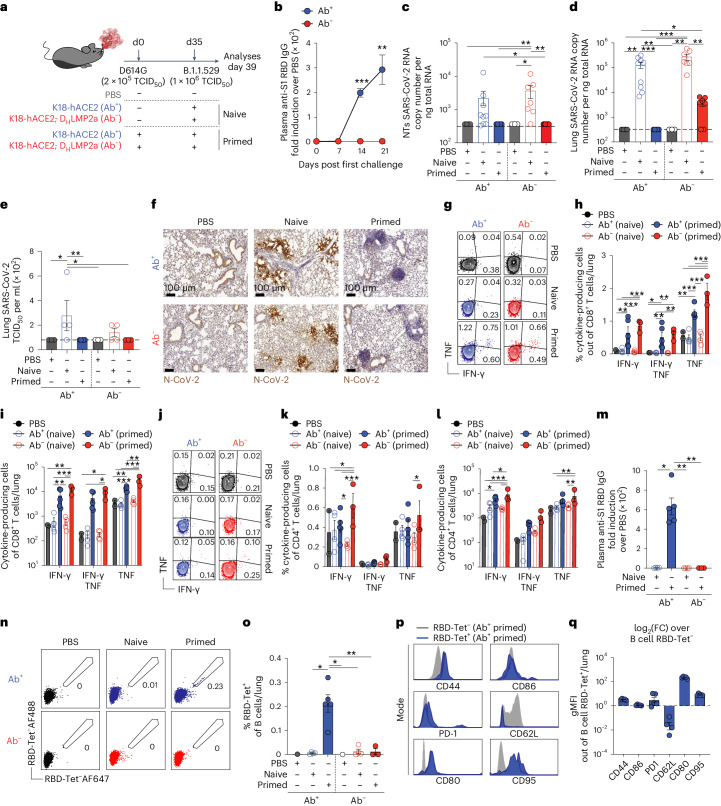
Antibody-independent protection in K18-hACE2 transgenic mice. **a**, Experimental setup. Ab^+^ (*n* = 5) and Ab^−^ (*n* = 3–7) K18-hACE2 mice were primed with 2 × 10^5^ TCID_50_ of SARS-CoV-2 D614G and rechallenged with a higher dose (1 × 10^6^ TCID_50_) of SARS-CoV-2 B.1.1.529 (Omicron). Ab^+^ (*n* = 4–9) and Ab^−^ (*n* = 4–7) naïve mice, unexposed to the primary challenge, were infected with 1 × 10^6^ TCID_50_ of SARS-CoV-2 B.1.1.529. PBS-exposed mice were used as controls. Blood was collected 7, 14 and 21 days after the first infection. Blood, lung, NT and mediastinal lymph node (mLN) were collected 4 days after rechallenge. **b**, Anti-S1 RBD IgG levels in the plasma after the first challenge. **c**,**d**, SARS-CoV-2 RNA in the NT (**c**) and lung (**d**). RNA values as copy number per ng of total RNA and the LOD as a dashed line. **e**, Viral titers in the lung were determined by TCID_50_. **f**, Immunohistochemical micrographs of lung sections from PBS-, naïve- and primed-Ab^+^ and Ab^−^ mice. N-SARS-CoV-2-positive cells in brown. Scale bars, 100 μm. **g**–**l**, Flow cytometry plots (**g** and **j**), frequency (**h** and **k**) and absolute number (**i** and **l**) of CD8^+^ T cells (**g**–**i**) or CD4^+^ T cells (**j**–**l**) expressing IFN-γ and TNF in the lungs upon in vitro stimulation with a pool of SARS-CoV-2 peptides. Plots pregated as live^+^/B220^−^/CD19^−^/CD4^−^/CD8^+^ (**g**–**i**) or CD8^−^/CD4^+^ (**j**–**l**). **m**, Anti-S1 RBD IgG levels in the plasma 4 days after rechallenge. **n**,**o**, Flow cytometry plots (**n**) and frequency (**o**) of RBD-specific B cells detected by RBD-tetramers in the lungs (pregated on live^+^/CD4^−^/CD8^−^/B220^+^/CD19^+^). **p**,**q**, Flow cytometry histogram (**p**) and geometric mean fluorescence intensity (gMFI) (**q**) of surface markers expressed by RBD-specific B cells in the lung of Ab^+^ primed mice. As control, B cells negative for RBD-tetramer staining (gray). gMFI as log_2_(fold change) over control B cells. Data are expressed as mean ± s.e.m. and are representative of at least two independent experiments. Data in **b**–**d** are pooled from two independent experiments. *P < 0.05, **P < 0.01, ***P < 0.001; Kruskal–Wallis test followed by uncorrected Dunn’s test; each comparison stands alone (**c**–**e**, **m** and **o**). Two-way ANOVA, Fisher’s LSD test (each comparison stands alone; **b**, **h**, **i**, **k** and **l**). LSD, least significant difference. Source data

A subset of the surviving Ab^−^ K18-hACE2 transgenic mice were subjected to a heterologous rechallenge with a higher dose (1 × 10^6^ TCID_50_) of aerosolized SARS-CoV-2 (B.1.1.529 (Omicron)) to evaluate cross-protection in the absence of humoral immunity (Fig. 1a). For comparative insights, we also studied previously infected Ab^+^ K18-hACE2 mice and naïve mice unexposed to the primary SARS-CoV-2 (D614G) challenge (Fig. 1a). Ab^+^ K18-hACE2 transgenic mice predictably mounted a robust immunoglobulin G (IgG) response toward the receptor-binding domain (RBD) contained within the S1 subunit of the spike protein (Fig. 1b) and effectively controlled viral replication so that no viral RNA was detected in the nasal turbinates (NTs) and lungs 4 days after heterologous rechallenge (Fig. 1c,d). Surprisingly, Ab^−^ K18-hACE2 transgenic mice, despite lacking an antibody response against SARS-CoV-2 (Fig. 1b), also effectively controlled viral replication upon heterologous rechallenge, as no viral RNA was detected in the NTs and was decreased by two logs in the lungs 4 days after rechallenge (Fig. 1c,d). Consistent with these results, infectious virus and SARS-CoV-2 nucleoprotein were detected in the lungs of naïve mice, whereas they were undetectable in the lungs of both Ab^+^ and Ab^−^ primed mice (Fig. 1e,f). In addition, immunohistochemistry and immunofluorescence staining of the lungs of both Ab^+^ and Ab^−^ primed mice revealed the presence of immune cell infiltrates, including both T cells and B cells (Fig. 1f and Extended Data Fig. 1d). Upon SARS-CoV-2 rechallenge, both Ab^+^ and Ab^−^ primed mice exhibited a significant increase in the frequency and absolute number of SARS-CoV-2-specific CD8^+^ and CD4^+^ T cells producing interferon-γ (IFN-γ) and/or tumor necrosis factor (TNF) upon in vitro stimulation with overlapping peptides covering the spike, membrane and nucleoprotein of SARS-CoV-2 (ref. ^31^; Fig. 1g–l). Yet, as expected, only primed Ab^+^ K18-hACE2 transgenic mice displayed increased levels of virus-specific antibodies in the plasma and activated RBD-specific B cells in the lungs and mediastinal lymph nodes (Fig. 1m–q and Extended Data Fig. 1e). Note that 4 days postinfection is too early to anticipate specific cellular or humoral responses in naïve mice exposed to SARS-CoV-2 for the first time (Fig. 1g–q and Extended Data Fig. 1e).

The findings collectively establish that even in the absence of antibodies, K18-hACE2 transgenic mice can resist a heterologous SARS-CoV-2 challenge effectively.

### A hyACE2 knock-in mouse supports SARS-CoV-2 infection

**Fig. 2:**
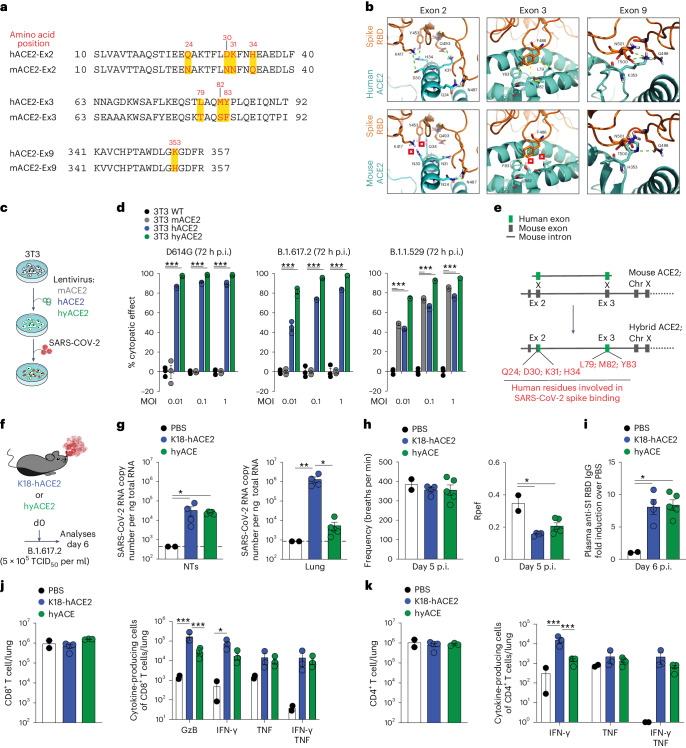
A hyACE2 knock-in mouse supports SARS-CoV-2 infection. **a**, Amino acid sequence of human (h)ACE2 and mouse (m)ACE2. In red are the eight residues involved in the interaction with the SARS-CoV-2 spike protein. **b**, Molecular modeling of the interaction between SARS-CoV-2 spike RBD (orange) and hACE2 or mACE2 (cyan). Crosses indicate the absence of interaction. Electrostatic and hydrophobic interactions in green and yellow dashed lines. **c**, Experimental setup. The 3T3 cells transduced with lentiviral vectors to express hACE2 (blue symbols), mACE2 (gray symbols) and a hybrid human/mouse (hy)ACE2 (green symbols) were infected with different concentrations of SARS-CoV-2. Nontransduced (WT) 3T3 cells as control. *n* = 3 biological replicates. **d**, Dose-dependent viral activity in 3T3 cells infected with SARS-CoV-2 D614G (left), B.1.617.2 (middle) or B.1.1.529 (right). Infection rates as a percentage of the virus-induced cytopathic effect 72 h after infection. Comparison with WT 3T3 cells. *n* = 3 biological replicates. **e**, Design of human/mouse hybrid *Ace2* allele. **f**, Experimental setup. K18-hACE2 transgenic mice (*n* = 4) and hyACE2 knock-in mice (*n* = 5) were infected with 5 × 10^5^ TCID_50_ of SARS-CoV-2 B.1.617.2 (Delta). PBS-exposed mice were used as controls (*n* = 2). Peripheral blood, lung and NT were analyzed 6 days after challenge. **g**, SARS-CoV-2 RNA in the NT (left) and lung (right). RNA values as copy number per ng of total RNA and the LOD as a dashed line. **h**, Respiratory frequency (left) and Rpef (right) were assessed by WBP 5 days postinfection (average over a 15-min data collection period). **i**, Anti-S1 RBD IgG levels in the plasma. **j**,**k**, Absolute number of total CD8^+^ T cells (**j**, left) and CD4^+^ T cells (**k**, left) and of cytokine-producing CD8^+^ cells (**j**, right) and CD4^+^ cells (**k**, right) in the lung on in vitro stimulation with a pool of SARS-CoV-2 peptides. Data are expressed as mean ± s.e.m. and are representative of at least two independent experiments. *P < 0.05, **P < 0.01, ***P < 0.001; Kruskal–Wallis test followed by uncorrected Dunn’s test; each comparison stands alone (**g**–**i**, **j** and **k** (left)). Two-way ANOVA, Tukey’s multiple comparison (**j** and **k** (right)); two-way ANOVA, Fisher’s LSD test (each comparison stands alone; **d**). Source data

To overcome the high mortality rate observed in Ab^−^ K18-hACE2 transgenic mice upon primary SARS-CoV-2 infection, we sought to establish a mouse model that remains susceptible to multiple SARS-CoV-2 variants while preserving physiological *Ace2* expression. While human and mouse *Ace2* share 82% sequence identity, differences in amino acids at the binding interface with the spike protein’s RBD are evident. Specifically, eight residues in human (h) ACE2 that interact with the SARS-CoV-2 spike protein^32^ differ in mice, categorized by their location in exons—exon 2 contains Q24 (N in mouse), D30 (N), K31 (N) and H34 (Q); exon 3 has L79 (T), M82 (S) and Y83 (F); exon 9 features K353 (H) (Fig. 2a). To understand the importance of these differences, we developed a molecular model, substituting the eight human-specific residues with their mouse equivalents, and juxtaposed this model ACE2 with the original hACE2 structure to identify differential interactions with the RBD (Fig. 2b). The hACE2–RBD interaction predominantly hinges on two polar contact networks and one hydrophobic region^32,33^. One polar network is represented by residues Q24, D30, K31 and H34, encoded by exon 2 of hACE2, that are engaged in electrostatic contacts with RBD residues N487, K417, Q493 and Y453, respectively (Fig. 2b). The different mACE2 residues might compromise two of four polar contacts (namely N30-K417 and Q34-Y453). The hydrophobic patch, composed of the L79, M82 and Y83 residues, encoded by exon 3 of hACE2, is responsible for interacting with F486 of SARS-CoV-2 RBD^34,35^ (Fig. 2b). In the mACE2 model, the contact between F83 (mACE2) and F486 (RBD) is maintained, while the polar residues T79 and S82 lose their ability to engage the spike protein through hydrophobic interactions. Residue K353, encoded by exon 9 of hACE2, forms hydrogen bonds with the polar amino acids Q498, T500 and N501 (ref. ^36^), but these interactions are not expected to be lost following a K-to-H substitution (Fig. 2b).

**Extended Data Fig. 2:**
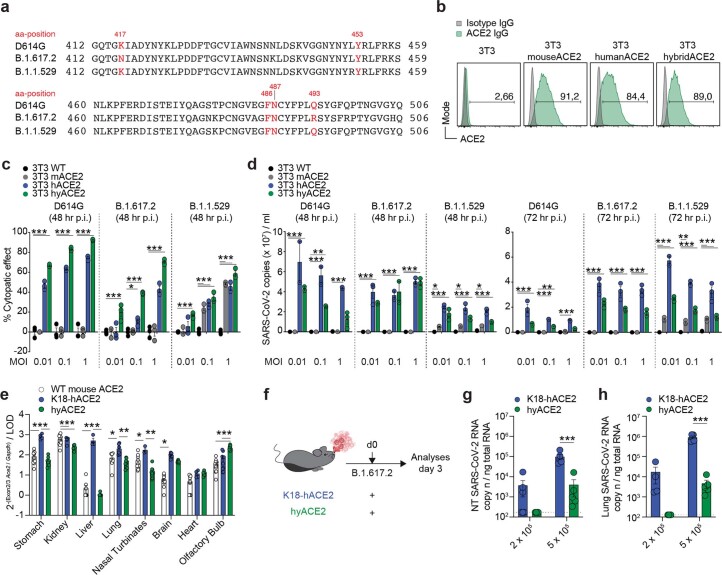
A novel human/mouse hybrid ACE2 knock-in mouse supports SARS-CoV-2 infection. (**a**) Amino acid sequence alignment of SARS-CoV-2 D614G, B.1.617.2 and B.1.1.529. The residues involved in the interaction with the human ACE2 are indicated in red. (**b**) Representative flow cytometry histograms representing the ACE2 expression by 3T3 cells and 3T3 cells transduced with murine ACE2, human ACE2 and hybrid ACE2. (**c, d**) SARS-CoV-2 titers in transduced 3T3 cells upon infection with D614G (left), B.1.617.2 (middle) or B.1.1.529 (right). In (**c**) titers were determined as percent of the virus-induced cytopathic effect evaluated 48 hours after infection. In (**d**) titers were determined by qPCR quantification of SARS-CoV-2 RNA in the supernatant 48 and 72 hours postinfection. *n* = 3 biological replicates. (**e**) Human ACE2 expression in the indicated organs from WT (gray), K18-hACE2 (blue) and hyACE2 (green) mice. Values were normalized to the reference gene *Gapdh* and expressed as fold increase over the limit of detection (LOD). *n* = 7-8 mice. (**f**) K18-hACE2 transgenic mice (*n* = 5) and hyACE2 knock-in mice (*n* = 5) were infected with a target dose of 2 ×10^5^ or 5 ×10^5^ TCID_50_ of SARS-CoV-2 B.1.617.2 through aerosol exposure. Lung and nasal turbinates (NT) were collected and analyzed 3 days postchallenge. (**g**, **h**) Quantification of SARS-CoV-2 RNA in the NT (**g**) and in the lung (**h**) of the indicated mice. *n* as indicated in (f). RNA values are expressed as copy number per ng of total RNA and the limit of detection is indicated as a dotted line. Data are expressed as mean ± SEM. Data are representative of at least 2 independent experiments. *p value < 0.05, **p value < 0.01, ***p value < 0.001; Two-way ANOVA, Fisher’s LSD test (Each comparison stands alone) (**c**–**e**, **g**, **h**). Source data

From our evaluation, seven critical amino acid substitutions across exons 2 and 3 in mACE2 emerged as paramount for RBD engagement. In contrast, the single amino acid variation in exon 9 appeared less consequential. Notably, three pivotal amino acids in hACE2 interaction, Y453, F486 and N487, remained consistent across several SARS-CoV-2 variants (that is, D614G, B.1.617.2 (Delta) and B.1.1.529 (Omicron); Extended Data Fig. 2a).

Shifting our focus to an experimental approach, we constructed lentiviral vectors to express hACE2, mACE2 and a hyACE2 where the N-terminal part of mACE2—encoded by exons 2 and 3—was replaced by the N-terminal part of hACE2, and therefore incorporated the seven essential human residues for RBD interaction. Testing these constructs on 3T3 cells, we assessed infection rates by several SARS-CoV-2 variants via cytopathic effects and RNA detection (Fig. 2c,d and Extended Data Fig. 2b–d). We found that cells expressing hACE2 and hyACE2 were effectively infected by all three variants, and, consistent with previous findings^34^, those expressing mACE2 were notably susceptible to the SARS-CoV-2 B.1.1.529 (Omicron) variant but not to the other variants (Fig. 2d and Extended Data Fig. 2c,d).

Furthering our investigation, after confirming hyACE2’s infection susceptibility akin to hACE2, we used CRISPR–Cas9 to replace the *mAce2* sequence with the *hyACE2* sequence (Fig. 2e). Notably, this knock-in replaces only part of exon 2 and all exon 3 in the mouse genome, with all the other genetic information, including introns and gene control regions, unchanged. Predictably, *Ace2* expression levels in tissues of hyACE2 mice matched those of wild-type (WT) mice but were considerably lower than those of K18-hACE2 transgenic mice (Extended Data Fig. 2e). Although hyACE2 mice showed detectable SARS-CoV-2 RNA after exposure (experimental setup shown in Fig. 2f), they needed a higher viral dose for infection (Extended Data Fig. 2f–h) and had reduced viral RNA in the lungs compared to K18-hACE2 mice (Fig. 2g). Even with this diminished viral replication in the lungs, hyACE2 mice displayed comparable pulmonary function impairment, as evidenced by changes in Rpef (an indicator of airway obstruction) measured using whole-body plethysmography (WBP; Fig. 2h). Additionally, both mouse groups demonstrated similar SARS-CoV-2-specific adaptive immune responses (Fig. 2i–k).

In conclusion, we have successfully generated a new knock-in mouse model, exhibiting minimal changes to the native *Ace2* gene, that both conserves physiological *Ace2* expression and exhibits gain-of-function vulnerability to multiple SARS-CoV-2 variants.

### Antibody-independent protection in hyACE2 mice

**Fig. 3:**
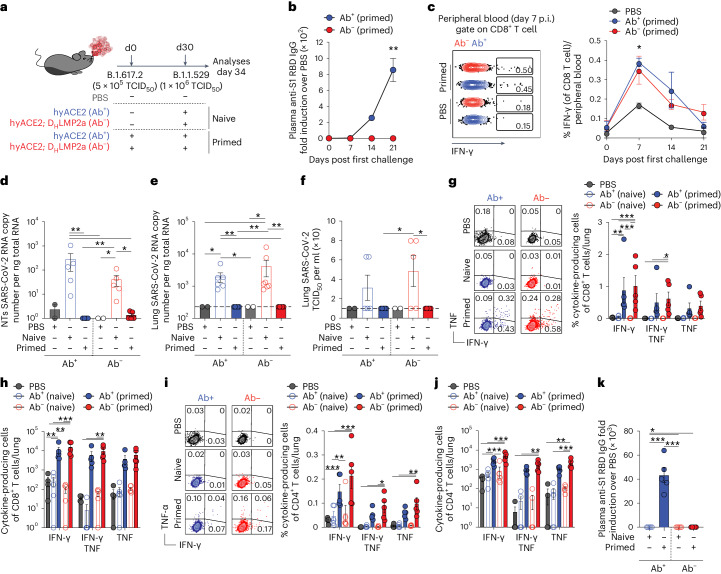
Antibody-independent protection in hyACE2 mice. **a**, Experimental setup. Ab^+^ (*n* = 5) and Ab^−^ (*n* = 5) hyACE2 knock-in mice were primed with 5 × 10^5^ TCID_50_ of SARS-CoV-2 B.1.617.2 (Delta) and rechallenged with a higher dose (1 × 10^6^ TCID_50_) of SARS-CoV-2 B.1.1.529 (Omicron). Ab^+^ (*n* = 5) and Ab^−^ (*n* = 7) naïve mice, unexposed to the primary challenge, were infected only with 1 × 10^6^ TCID_50_ of SARS-CoV-2 B.1.1.529 (Omicron). As control, PBS-exposed mice. Blood was collected 7, 14 and 21 days after the first infection. Blood, lung, NT and mLN were analyzed 4 days after rechallenge. **b**, Anti-S1 RBD IgG levels in the plasma after the first challenge. Number (*n*) of mice as in **a**. **c**, Dot plots (left) and frequency (right) of IFN-γ-producing CD8^+^ T cells in the peripheral blood after the first challenge. Number (*n*) of mice as in **a**. **d**,**e**, SARS-CoV-2 RNA in the NT (**d**) and lung (**e**). RNA values as copy number per ng of total RNA and the LOD as a dashed line. **f**, Viral titers in the lung were determined by TCID_50_. **g**,**i**, Flow cytometry plots (left) and frequency (right) of CD8^+^ T cells (**g**) or CD4^+^ T cells (**i**) expressing IFN-γ and TNF in the lungs upon in vitro stimulation with a pool of SARS-CoV-2 peptides. Plots pregated as live^+^/B220^−^/CD19^−^/CD4^−^/CD8^+^ cells (**g**) or CD8^−^/CD4^+^ (**i**). **h**,**j**, Absolute number of cytokine-producing CD8^+^ T cells (**h**) or CD4^+^ T cells (**j**). **k**, Anti-S1 RBD IgG levels in the plasma 4 days after rechallenge. Data are expressed as mean ± s.e.m. and are representative of at least two independent experiments. *P < 0.05, **P < 0.01, ***P < 0.001; Kruskal–Wallis test followed by uncorrected Dunn’s test; each comparison stands alone (**d**–**f** and **k**). Two-way ANOVA, Fisher’s LSD test (each comparison stands alone; **b**, **c**, **g**–**j**). Source data

**Extended Data Fig. 3:**
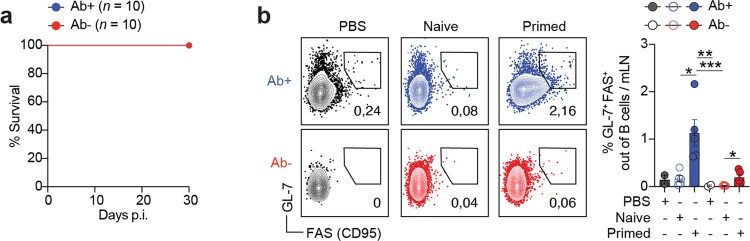
Previously infected, antibody-deficient hyACE2 knock-in mice are protected against heterologous SARS-CoV-2 re-challenge. (**a**) Survival curve of Ab^+^ (*n* = 10, blue line) and Ab^−^ hyACE2 upon SARS-CoV-2 infection (*n* = 10, red line). Mice were exposed to 5 × 10^5^ TCID_50_ of SARS-CoV-2 B.1.617.2, as described in Fig. 3. (**b**) Representative flow cytometry plots (left) and frequency (right) of GL-7^+^ FAS^+^ B cells in the mLN of indicated mice 4 days post re-challenge (pre-gated on live^+^/CD4^−^/CD8^−^/B220^+^/CD19^+^ cells). *n* as indicated in Fig. 3a. Data are expressed as mean ± SEM. Data are representative of at least 2 independent experiments. *p value < 0.05, **p value < 0.01, ***p value < 0.001; Kruskal-Wallis test followed by uncorrected Dunn’s test, each comparison stands alone (**b**). Source data

We bred the newly developed hyACE2 knock-in mice with D_H_LMP2a mice to generate Ab^−^ animals. Unlike Ab^−^ K18-hACE2, 100% of these Ab^−^ hyACE2 knock-in mice survived aerosol exposure to SARS-CoV-2 variant B.1.617.2 (Delta; 5 × 10^5^ 50% tissue culture infectious dose (TCID_50_); Fig. 3a and Extended Data Fig. 3a). Predictably, only Ab^+^ but not Ab^−^, hyACE2 knock-in mice mounted an anti-RBD-specific IgG response (Fig. 3b). Yet, both Ab^+^ and Ab^−^ hyACE2 mice developed equivalent SARS-CoV-2-specific CD8^+^ T cell responses, peaking 7 days postinfection (Fig. 3c). At day 30 after primary infection, we exposed both groups of mice to 10^6^ TCID_50_ of a different SARS-CoV-2 variant (B.1.1.529 (Omicron); Fig. 3a). For comparison, we also infected naïve Ab^+^ and Ab^−^ hyACE2 mice with the same B.1.1.529 (Omicron) dose (Fig. 3a). While naïve mice showed signs of successful SARS-CoV-2 infection 4 days postinfection with the B.1.1.529 (Omicron) variant, including viral RNA and infectious virus in the NTs and lungs (Fig. 3d–f), previously exposed mice did not exhibit detectable viral RNA or infectious virus (Fig. 3d–f). Consistent with earlier results (Fig. 1), this viral control in both Ab^+^ and Ab^−^ primed mice corresponded with the presence in the lungs of SARS-CoV-2-specific CD8^+^ and CD4^+^ T cells that produced IFN-γ and/or TNF upon in vitro cognate peptide stimulation (Fig. 3g–j). It is worth noting that 4 days postinfection is premature to expect specific cellular or humoral responses in naïve mice encountering B.1.1.529 (Omicron) for the first time (Fig. 3g–k). Aligning with the significant anti-RBD antibody titer in Ab^+^ hyACE2 mice post rechallenge with the B.1.1.529 (Omicron) variant, we detected germinal center (GL7^+^ FAS^+^) B cells in their mediastinal lymph nodes (Extended Data Fig. 3b).

These data, obtained with a newly generated independent mouse model, confirm an antibody-independent protective mechanism against heterologous SARS-CoV-2 challenge conferred by a previous infection.

### Antibody-independent protection against severe disease

**Fig. 4:**
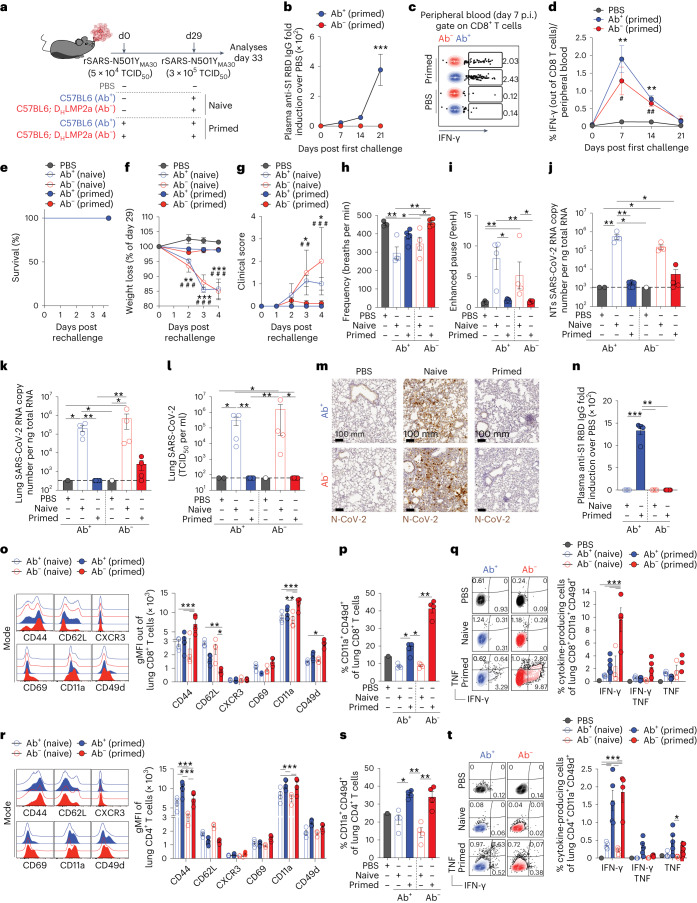
Antibody-independent protection against severe disease. **a**, Experimental setup. Ab^+^ (*n* = 5) and Ab^−^ (*n* = 4) C57BL/6 mice were primed with 5 × 10^4^ TCID_50_ of rSARS-N501Y_MA30_ and rechallenged with a higher dose (3 × 10^5^ TCID50) of rSARS-N501Y_MA30_. Ab^+^ (*n* = 4) and Ab^−^ (*n* = 4) naïve mice, unexposed to the primary challenge, were infected with 3 × 10^5^ TCID_50_ of rSARS-N501Y_MA30_. PBS-exposed mice were used as controls. Blood was collected 7, 14 and 21 days after the first infection. Blood, lung and NT were analyzed 4 days after rechallenge. **b**, Anti-S1 RBD IgG levels in the plasma after the first challenge. Number (*n*) of mice as in **a**. **c**,**d**, Dot plots (**c**) and frequency (**d**) of IFN-γ-producing CD8^+^ T cells in the blood after the first challenge. Asterisk indicates Ab^+^ (primed) compared to PBS; hash indicates Ab^−^ (primed) compared to PBS. Number (*n*) of mice as in **a**. **e**, Survival curve after the rechallenge. **f**, Mouse body weight after the rechallenge as a percentage of weight relative to day 29. Asterisk and hash indicate Ab^+^ and Ab^−^ compared to PBS, respectively. **g**, Clinical score. Number (*n*) of mice as in **a**. **h**,**i**, Respiratory frequency (**h**) and PenH (**i**) were assessed by WBP 3 days after rechallenge (average over a 15-min data collection period). **j**,**k**, SARS-CoV-2 RNA in the NT (**j**) and lung (**k**). RNA values as copy number per ng of total RNA and the LOD as a dashed line. **l**, Viral titers in the lung were determined by TCID_50_. **m**, Immunohistochemical micrographs of lung sections from PBS-, naïve- and primed-Ab^+^ and Ab^−^ mice. N-SARS-CoV-2-positive cells in brown. Scale bars, 100 μm. **n**, Anti-S1 RBD IgG levels in the plasma 4 days after rechallenge. **o**,**r**, Flow cytometry histogram and gMFI of surface markers expressed by CD8^+^ T cells (**o**) and CD4^+^ T cells (**r**) in the lung. **p**,**s**, Frequency of CD11a, CD49d CD8^+^ T cells (**p**) and CD4^+^ T cells (**s**) in the lung. **q**,**t**, Plots and frequency of CD11a^+^, CD49d^+^ CD8^+^ T cells (**q**) or CD4^+^ T cells (**t**) expressing IFN-γ^+^/TNF^+^ upon in vitro stimulation with a pool of SARS-CoV-2 peptides. Plots pregated as live^+^/B220^−^/CD19^−^. Data are expressed as mean ± s.e.m. and are representative of at least two independent experiments. *,^#^*P* < 0.05, **,^##^*P* < 0.01, ***,^###^*P* < 0.001; Kruskal–Wallis test followed by uncorrected Dunn’s test; each comparison stands alone (**h**–**l**, **n**, **p** and **s**). Two-way ANOVA, Fisher’s LSD test (each comparison stands alone; **b**, **d**, **f**, **g**, **o**, **q**, **r** and **t**). Source data

**Extended Data Fig. 4:**
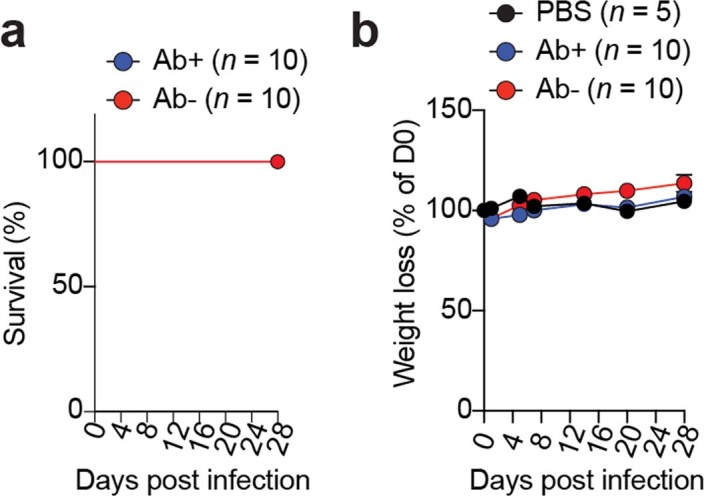
Antibody-independent protection against severe disease. (**a**, **b**) Survival curve (**a**) and body weight loss (**b**) of Ab^+^ (*n* = 10, blue symbols) and Ab^−^(*n* = 10, red symbols) C57BL/6 mice upon SARS-CoV-2 infection. Mice were exposed to 5 × 10^4^ TCID_50_ of aerosolized rSARS-N501Y_MA30_, as described in Fig. 4. Data are expressed as mean ± SEM. Data are representative of at least 2 independent experiments. Source data

Aerosol exposure of K18-hACE2 transgenic as well as hyACE2 knock-in mice to SARS-CoV-2 mimics mild COVID-19 in humans. To probe for the possibility of antibody-independent protection from severe disease, we used a particularly virulent, mouse-adapted SARS-CoV-2 strain, rSARS-N501Y_MA30_ (ref. ^37^). When Ab^+^ and Ab^−^ C57BL/6 mice were infected with a sublethal (5 × 10^4^ TCID_50_) dose of this strain (Fig. 4a and Extended Data Fig. 4a,b), we predictably detected virus-specific antibodies in the plasma of Ab^+^, but not Ab^−^, mice by day 7 postinfection (Fig. 4b). Yet, both Ab^+^ and Ab^−^ mice showed virus-specific IFN-γ^+^ CD8^+^ T cells in their bloodstream (Fig. 4c,d). After this initial exposure, we rechallenged these primed mice with a higher (3 × 10^5^ TCID_50_) dose of rSARS-N501Y_MA30_ on day 29. Naïve mice, exposed to rSARS-N501Y_MA30_ for the first time, displayed significant body weight loss and a severe clinical score starting 2 days postinfection (Fig. 4f,g). In stark contrast, both primed Ab^+^ and Ab^−^ mice survived the infection, remained stable in weight and exhibited no disease signs (Fig. 4e–g). We assessed their respiratory function using WBP on day 3 after rechallenge. Naïve mice, both Ab^+^ and Ab^−^, showed reduced respiratory frequency and heightened PenH, indicative of increased airway resistance (Fig. 4h,i). Yet, these respiratory metrics were unchanged in primed Ab^+^ and Ab^−^ mice (Fig. 4h,i). Furthermore, while the NTs and lungs of naïve mice contained abundant viral RNA and infectious virus, primed mice from both groups displayed, at most, scant traces of RNA (Fig. 4j–l), a finding reinforced by immunohistochemical staining for the SARS-CoV-2 nucleoprotein (Fig. 4m). Post rechallenge, Ab^+^ primed, but not naïve, mice showed high anti-RBD antibody titers by day 4 (Fig. 4n). Notably, both CD4^+^ and CD8^+^ T cells from the lungs of primed mice of both categories displayed characteristics of tissue-resident memory cells (T_RM_). These T_RM_ are identifiable by their downregulated CD62L and upregulated CD44, CD11a (LFA-1) and CD49d (VLA-1; Fig. 4o,r). Primed mice had a considerably higher frequency of these CD11a^+^ CD49d^+^ T cells compared to naïve or noninfected controls (Fig. 4p,s). Additionally, these T_RM_ expressed IFN-γ and/or TNF upon in vitro peptide stimulation (Fig. 4q,t).

Using a third independent mouse model, our findings confirm a mechanism of protection against severe COVID-19 that operates independently of antibodies.

### Antibody-independent protection conferred by prior mRNA vaccination

**Fig. 5:**
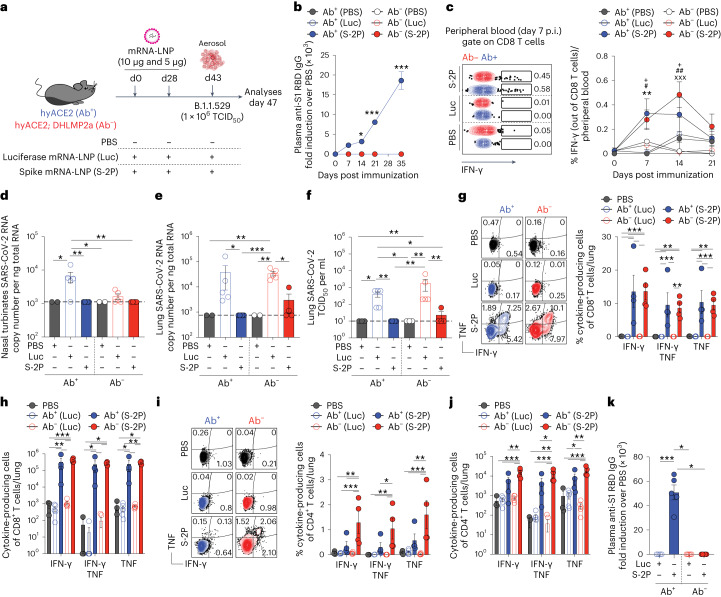
Antibody-independent protection conferred by prior mRNA vaccination. **a**, Experimental setup. Ab^+^ (*n* = 9) and Ab^−^ (*n* = 9) hyACE2 knock-in mice were immunized with SARS-CoV-2 full-length spike (S-2P) or firefly luciferase (Luc) mRNA-LNP. Fifteen days after the boost (day 43), mice were exposed to a heterologous challenge of 1 × 10^6^ TCID_50_ of SARS-CoV-2 B.1.1.529 (Omicron). As control, nonimmunized mice were exposed to PBS. Blood was collected 7, 14, 21 and 35 days after the immunization. Blood, lung, NT, mLN and spleens were analyzed 4 days postinfection. **b**, Anti-S1 RBD IgG levels in the plasma after the immunization. Number (*n*) of mice as in **a**. **c**, Dot plots (left) and frequency (right) of IFN-γ-producing CD8^+^ T cells in the peripheral blood after the first dose of immunization. Asterisk indicates Ab^+^ (S-2P) compared to Ab^+^ (Luc); hash indicates Ab^−^(S-2P) compared to Ab^+^ (Luc); + symbol indicates Ab^+^ (S-2P) compared to Ab^−^ (Luc); x symbol indicates Ab^−^ (S-2P) compared to Ab^−^(Luc). Number (*n*) of mice as in **a**. **d**,**e**, SARS-CoV-2 RNA in the (**d**) NT and (**e**) lung. RNA values as copy number per ng of total RNA and the LOD as a dashed line. **f**, Viral titers in the lung were determined by TCID_50_. **g**,**i** Representative flow cytometry plots (left) and frequency (right) of CD8^+^ T cells (**g**) or CD4^+^ T cells (**i**) expressing IFN-γ and TNF in the lungs on in vitro stimulation with a pool of SARS-CoV-2 peptides. Plots were pregated as live^+^/B220^−^/CD19^−^/CD4^−^/CD8^+^ cells (**g**) or CD8^−^/CD4^+^ cells (**i**). **h**,**j**, Absolute number of cytokine-producing CD8^+^ T cells (**h**) or CD4^+^ T cells (**j**). **k**, Anti-S1 RBD IgG levels in the plasma 4 days postinfection. Data are expressed as mean ± s.e.m. and are representative of at least two independent experiments. *,^#^,^x^,^+^*P* < 0.05; **,^##^,^xx^,^++^*P* < 0.01; ***,^###^,^xxx^,^+++^*P* < 0.001; Kruskal–Wallis test followed by uncorrected Dunn’s test; each comparison stands alone (**d**–**f** and **k**). Two-way ANOVA, Fisher’s LSD test (each comparison stands alone; **b**, **c**, **g**–**j**). Source data

**Extended Data Fig. 5:**
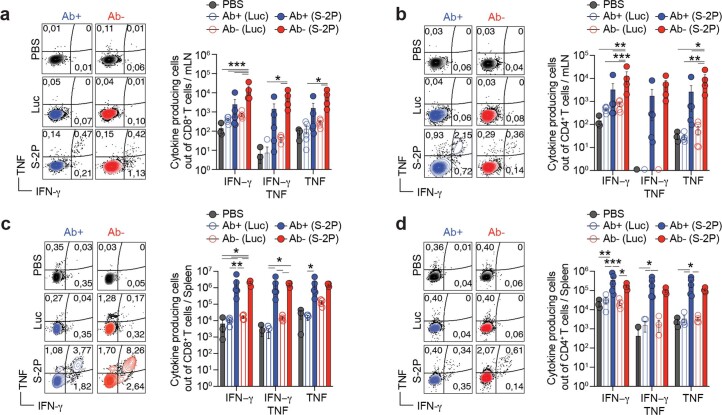
Antibody-independent protection against heterologous SARS-CoV-2 infection conferred by prior mRNA vaccination. (**a**–**d**) Frequency of CD8^+^ T cells (left) and CD4^+^ T cells (right) in the mediastinal lymph nodes (**a**, **b**) and spleens (**c**, **d**) of the indicated mice 4 days postinfection. Experimental setup is described in Fig. 5, and n of mice is indicated in Fig. 5a. Data are expressed as mean ± SEM. Data are representative of at least 2 independent experiments. *p value < 0.05, **p value < 0.01, ***p value < 0.001; Two-way ANOVA, Fisher’s LSD test (Each comparison stands alone) (**a**–**d**). Source data

We next set out to determine the potential of mRNA vaccination to protect against SARS-CoV-2 infection independent of humoral immunity. To this end, we immunized both Ab^+^ and Ab^−^ hyACE2 mice with lipid nanoparticle (LNP)-encapsulated, nucleoside-modified mRNA vaccines. These vaccines encoded either the SARS-CoV-2 Wuhan full-length spike with K986P and V987P amino acid substitutions (S-2P)^38^ or the firefly luciferase (Luc), which served as a negative control (Fig. 5a). RBD-specific IgG predictably appeared in the serum of Ab^+^ hyACE2 knock-in mice, but not in the Ab^−^ counterparts (Fig. 5b). Regardless of antibody presence, both mouse groups developed SARS-CoV-2-specific CD8^+^ T cells that expressed IFN-γ^+^ upon in vitro cognate peptide stimulation (Fig. 5c). Following two doses of LNP-mRNA vaccines, hyACE2 mice were exposed to a heterologous challenge with the SARS-CoV-2 variant B.1.1.529 (Omicron; Fig. 5a). Echoing our earlier findings (Fig. 3), both Ab^+^ and Ab^−^ vaccinated mice successfully limited viral replication in their NTs and lungs (Fig. 5d–f). They also exhibited comparable levels of SARS-CoV-2-specific IFN-γ^+^ and/or TNF^+^ CD8^+^ T and CD4^+^ T cells across the lungs, mediastinal lymph nodes and spleens (Fig. 5g–j and Extended Data Fig. 5). As expected, anti-RBD antibody titers 4 days after challenge were detected only in Ab^+^ mice vaccinated with S-2P mRNA-LNP, but not with Luc mRNA-LNP (Fig. 5k).

In conclusion, these findings underscore that prior mRNA vaccination can offer protection against a heterologous SARS-CoV-2 challenge, even in the absence of antibodies.

### Antibody-independent protection via T cells and IFN-γ

**Fig. 6:**
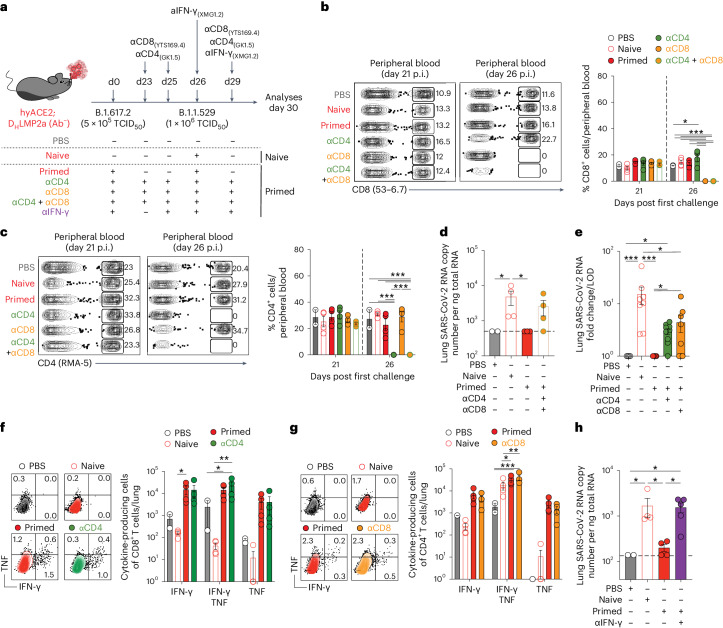
Antibody-independent protection via T cells and IFN-γ. **a**, Experimental setup. Ab^−^ hyACE2 knock-in mice were primed with 5 × 10^5^ TCID_50_ of SARS-CoV-2 B.1.617.2 (Delta) and rechallenged with a higher dose (1 × 10^6^ TCID_50_) of SARS-CoV-2 B.1.1.529 (Omicron). Ab^−^ (*n* = 4) naïve mice, unexposed to the primary challenge, were infected with 1 × 10^6^ TCID_50_ of SARS-CoV-2 B.1.1.529 (Omicron). A group of primed mice was injected intravenously with anti-CD4 (*n* = 4), or anti-CD8 (*n* = 4), or the combination of anti-CD4 and anti-CD8 (*n* = 4) depleting antibodies 2 (day 23) and 1 day (day 25) before re-infection and 3 days later (day 29). A group of primed mice was injected intravenously with anti-IFN-γ (*n* = 5) blocking antibodies 4 h before and 3 days after (day 29) the re-infection. PBS-exposed mice were used as controls. Blood was collected 21 and 26 days after the first infection. Lung and mLN were analyzed 4 days after rechallenge. **b**,**c**, Dot plots (left) and frequency (right) of CD8^+^ T cells (**b**) and CD4^+^ T cells (**c**) in the peripheral blood after the first challenge. **d**, SARS-CoV-2 RNA in the lung. RNA values as copy number per ng of total RNA and the LOD as a dashed line. **e**, SARS-CoV-2 RNA in the lung. RNA values as fold change of the RNA copy number per ng of total RNA over the LOD. Data are pooled from two independent experiments. **f**,**g**, Flow cytometry plots (left) and absolute number (right) of CD8^+^ T cells (**f**) or CD4^+^ T cells (**g**) expressing IFN-γ and TNF in the lungs upon in vitro stimulation with a pool of SARS-CoV-2 peptides. Plots pregated as live^+^/B220^−^/CD19^−^/CD4^−^/CD8^+^ cells (**f**) or CD8^−^/CD4^+^ (**g**). **h**, SARS-CoV-2 RNA in the lung. RNA values as copy number per ng of total RNA and the LOD as a dashed line. Data are expressed as mean ± s.e.m. and are representative of two independent experiments. *P < 0.05, **P < 0.01, ***P < 0.001; Kruskal–Wallis test followed by uncorrected Dunn’s test; each comparison stands alone (**d**, **e** and **h**). Two-way ANOVA, Fisher’s LSD test (each comparison stands alone; **b**, **c**, **f** and **g**). Source data

**Extended Data Fig. 6:**
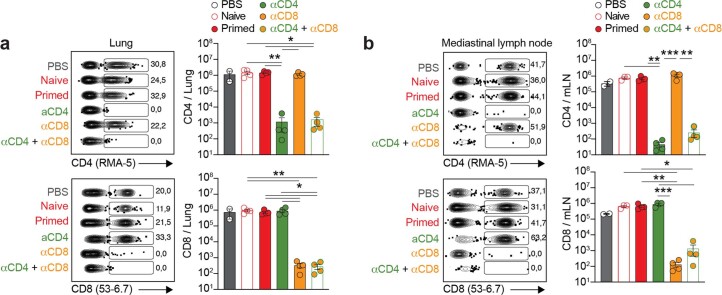
T cells and IFN-γ are key players in the antibody-independent protection against heterologous SARS-CoV-2 challenge. (**a**, **b**) Representative dot plots (left) and frequency (right) of CD4^+^ T cells (upper panel) and CD8^+^ T cells (lower panel) in the lungs (**a**) and mediastinal lymph nodes (**b**) of the indicated mice 4 days post re-challenge. Experimental setup is described in Fig. 6, and n of mice is indicated in Fig. 6a. Data are expressed as mean ± SEM. Data are representative of at least 2 independent experiments. *p value < 0.05, **p value < 0.01, ***p value < 0.001; Kruskal-Wallis test followed by uncorrected Dunn’s test, each comparison stands alone (**a**, **b**). Source data

We next aimed to uncover the mechanism driving the observed antibody-independent protection against heterologous SARS-CoV-2 challenge following a prior infection. In our experimental setup, Ab^−^ hyACE2 knock-in mice were exposed to the aerosolized SARS-CoV-2 variant B.1.617.2 (Delta; Fig. 6a). On the 23rd day postinfection, some mice underwent depletion of CD4^+^ T cells, CD8^+^ T cells or both, before being challenged with the SARS-CoV-2 variant B.1.1.529 (Omicron). As anticipated, our antibody-mediated depletion efficiently eliminated the target cell types from the peripheral blood, the lungs and the mediastinal lymph nodes (Fig. 6b,c and Extended Data Fig. 6a,b).

**Extended Data Fig. 7:**
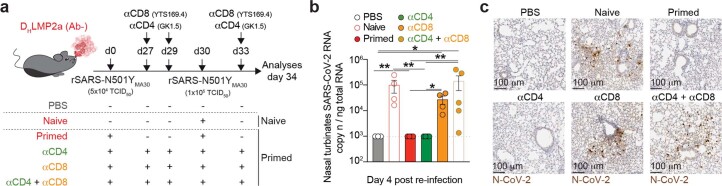
Critical role of CD8^+^ T cells in severe disease. (**a**) Experimental setup. Ab^-^ D_H_LMP2a mice were infected with a target dose of 5 × 10^4^ TCID_50_ of rSARS-CoV-2-N501Y_MA30_ through aerosol exposure. Thirty days after infection, primed mice were exposed to a heterologous challenge with a target dose of 1x10^5^ TCID_50_ of rSARS-CoV-2-N501Y_MA30_. Ab^−^ (*n* = 4) naïve mice unexposed to the primary challenge were infected with 1x10^5^ TCID_50_ of rSARS-CoV-2-N501Y_MA30_. A group of primed mice was injected intravenously with anti-CD4 (*n* = 4), or anti-CD8 (*n* = 4), or the combination of anti-CD4 and anti-CD8 (*n* = 5) depleting antibodies three (day 27) and one day (day 29) prior to re-infection and three days later (day 33). Non-infected mice exposed to aerosolized PBS were used as control. Analyses were performed 4 days post re-challenge. (**b**) Quantification of SARS-CoV-2 RNA in the nasal turbinates of the indicated mice. *n* as indicated in **a**. RNA values are expressed as copy number per ng of total RNA and the limit of detection is indicated as a dotted line. (**c**) Representative immunohistochemical micrographs of lung sections from indicated mice. N-SARS-CoV-2 positive cells are depicted in brown. Scale bars, 100 μm. Data are expressed as mean ± SEM and are representative of two independent experiments. *p value < 0.05, **p value < 0.01; Kruskal-Wallis test followed by uncorrected Dunn’s test, each comparison stands alone (**b**). Source data

As expected, naïve mice displayed evidence of successful SARS-CoV-2 infection, marked by the presence of viral RNA in the lungs 4 days postinfection (Fig. 6d). In contrast, mice previously exposed to the virus showed no detectable viral RNA upon being challenged with the B.1.1.529 (Omicron) variant (Fig. 6d). However, in primed mice, the depletion of both CD4^+^ and CD8^+^ T cells significantly compromised protection, leading to viral replication rates that were comparable to those observed in naïve mice (Fig. 6d). Regarding the contributions of CD4^+^ and CD8^+^ T cells to protection, our data, derived from a large set of experiments under diverse conditions, support the conclusion that both CD4^+^ and CD8^+^ T cells are instrumental for antibody-independent protection against heterologous SARS-CoV-2 challenge. While our results underscore the critical role of CD8^+^ T cells in managing high-dose infection or severe disease (Extended Data Fig. 7), they also highlight the substantial and complementary contribution of CD4^+^ T cells, especially in scenarios of low-dose infection or mild disease (Fig. 6e), with both subsets collaboratively mediating protection. It is worth emphasizing that depleting either CD4^+^ or CD8^+^ T cells did not influence the emergence of SARS-CoV-2-specific T cell responses for the remaining T cell type (Fig. 6f,g).

Additionally, our study delved into the mechanisms underlying T cell-mediated, antibody-independent protection. We used Ab^−^ hyACE2 knock-in mice exposed to the aerosolized SARS-CoV-2 variant B.1.617.2 (Delta) and treated them with anti-IFN-γ blocking antibodies before and 3 days after heterologous challenge with the SARS-CoV-2 variant B.1.1.529 (Omicron). The neutralization of IFN-γ significantly impaired the protective response, resulting in viral replication rates akin to those seen in naïve mice (Fig. 6h).

In summary, our findings pinpoint T cells and IFN-γ as pivotal in the antibody-independent protection against heterologous SARS-CoV-2 challenges following initial infection.

## Discussion

In this study, we systematically investigated the efficacy of T cell-mediated protection against heterologous SARS-CoV-2 challenges in the context of an absent humoral response. Using three independent animal models, including a new hybrid human/mouse ACE2 knock-in mouse, the evidence presented herein unambiguously indicates that mice lacking surface-expressed and circulating immunoglobulins—yet retaining functional B cells and intact secondary lymphoid organs—are capable of resisting heterologous SARS-CoV-2 challenges post prior infection or mRNA vaccination. Notably, our data reveal that memory T cells are effective not only in reducing pathology but also in substantially curtailing early viral replication. Our findings affirm that both CD4^+^ and CD8^+^ T cells are crucial in mediating this antibody-independent defense, with IFN-γ having a substantial role in this protection mechanism. The exact pathways through which IFN-γ exerts its antiviral effects are yet to be fully elucidated. However, it aligns with a wide spectrum of literature^39,40^ suggesting this cytokine’s role in preventing infection, activating antiviral states in infected cells, enhancing antigen processing and presentation and modulating the induction, amplification, recruitment and effector functions of various immune cells.

The hybrid human/mouse ACE2 knock-in model introduced herein contributes substantially to the extant limited array of mouse models capable of physiological ACE2 expression under the regulation of endogenous *Ace2* promoter elements, which are susceptible to multiple SARS-CoV-2 variants. This model exhibits distinct attributes concerning the number and localization of substituted amino acids and the iterations of CRISPR–Cas9-mediated editing, setting it apart from previously established models^41–44^. Subsequent comparative studies are imperative for a rigorous assessment of infection susceptibility and disease progression across these disparate models.

This study invites us to re-evaluate and refine the prevailing immunological paradigm concerning SARS-CoV-2. Current vaccine strategies overwhelmingly focus on generating potent neutralizing antibodies. Given the transient lifespan of such antibodies and the burgeoning threat of evasion by viral variants, the persistent efficacy of T cell responses challenges the notion of periodic vaccine updates and regular boosting. It is pertinent to mention that, unlike neutralizing antibodies, T cell responses have been documented to exhibit long-term durability following β-coronavirus infections^45^. Additionally, T cells recognize a broad epitopic landscape largely conserved despite mutations in emergent SARS-CoV-2 variants^46,47^.

While our investigation underscores the pivotal role of T cells in an antibody-independent milieu, it coheres with existing literature that elucidates T cell-mediated protective mechanisms in various animal models and human studies^12,48^. Individuals with primary or drug-induced B cell deficiencies may exhibit heightened T cell responses following SARS-CoV-2 infection or vaccination^49^. This enhanced T cell immunity could contribute to lower rates of hospitalization and less severe disease upon subsequent infections with SARS-CoV-2 (ref. ^49^).

It is essential to clarify that, although this study highlights the critical role of T cells, in no way it minimizes the importance of neutralizing antibodies. Within the multifaceted immunological framework, a synergistic action between antibodies and T cells is likely requisite for optimal protective immunity. Studies in mice and humans have shown that a coordinated and early activation of both humoral and cellular adaptive immunity is associated with effective viral control and the occurrence of minimal immunopathology^13,50^.

In conclusion, as endeavors to decipher the complex immunological landscape of SARS-CoV-2 accelerate, an integrative understanding of immune responses is crucial. The findings presented herein suggest that T cells can furnish robust immunity against heterologous SARS-CoV-2 challenges, even in the absence of humoral responses. This nuanced understanding should inform future vaccine development strategies and therapeutic interventions, providing a more holistic approach to tackling this pathogen.

## Methods

### Molecular modeling

Pairwise sequence alignment between human (h) and mouse (m)ACE2 was carried out by NCBI BLAST+ and EMBOSS Needle online tools. The cryo-EM resolved structure^32^ (Protein Data Bank (PDB) code: 6M17) containing the dimer of SARS‐CoV‐2 spike RBD coupled to hACE2 and sodium-dependent neutral amino acid transporter B(0)AT1 was used as the starting point for the modeling. The complex is composed of two SARS‐CoV‐2 RBD (chains E and F), the full-length hACE2 (chains B and D) and two B(0)AT1 (chains A and D). Chain B hACE2 in complex with chain E SARS‐CoV‐2 spike RBD was used for human-to-mouse mutagenesis modeling.

The residues of hACE2 interacting with the RBD were mutated in silico with the ‘Mutate residue’ tool available in Maestro 9.9 (Schrodinger Suite) to residues that are different in mACE2—specifically, exon 2-encoded residues Q24 (N in mouse), D30(N), K31(N) and H34(Q); exon 3-encoded residues L79(T), M82(S) and Y83(F); and exon 9-encoded residue K353(H). The Amber ff14SB force field was used^51^. The model was relaxed by short minimization runs of 1,000 steps, in implicit solvent using the generalized Born model, using the conjugate gradient energy method minimization algorithm (convergence criterion 1.0 × 10^−4^ kcal mol^−1^ Å). This allowed us to show that replacement of the seven N-terminal residues that contact the RBD would preclude productive interactions, while replacement of K353 with H would most likely be tolerated. For this reason, the entire N-terminal end of mACE2 was replaced in silico with the N-terminal part of hACE2, encoded in exons 2 and 3 of the *hACE2* gene. The model of hyACE2 did not show clashes between the human and the mouse portions and appeared capable of forming all critical contacts with the virus RBD.

### Cell lines

Human (h)ACE2, mouse (m)ACE2 or hybrid human/mouse (hy)ACE2 expression in 3T3 cells (ATCC CRL-1658) was obtained by lentiviral transduction. ACE2 receptor expression was confirmed and quantified by fluorescence-activated cell sorting (FACS) analysis using an anti-ACE2 antibody that cross-reacts with both mouse and hACE2 (Bio-Techne, AF933). Cells were maintained at 37 °C and 5% CO_2_ in Dulbecco’s Modified Eagle Medium (DMEM) supplemented with 10% FBS, 1% Minimum Essential Medium (MEM) nonessential amino acids, 100 U ml^−1^ penicillin, 100 µg ml^−1^ streptomycin, 2 mM glutamine and 250 μg ml^−1^ hygromycin (Gibco).

VERO-E6 (ATCC CRL-1587) were maintained at 37 °C and 5% CO_2_ in DMEM supplemented with 10% FBS, 1% MEM nonessential amino acids, 100 U ml^−1^ penicillin, 100 µg ml^−1^ streptomycin and 2 mM glutamine.

### Plasmids

pLENTI vectors expressing hACE2 and mACE2 were obtained by PCR cloning of ACE2 open reading frame from pcDNA3.1-hACE2 (provided by F. Li; Addgene, 145033) and pscALPSpuro-MmACE2 (provided by J. Luban; Addgene, 15808) under the cytomegalovirus (CMV) promoter of the pLenti-CMV-GFP-Hygro (provided by E. Campeau and P. Kaufman; Addgene, 17446) after green fluorescent protein (GFP) excision. The pLENTI vector expressing hyACE2 was constructed by replacing the mACE2 coding sequence with the chemically synthesized sequence (2.5 kb; Genewiz) coding for hyACE2 (where mouse amino acid codons downstream from the leader peptide in exon 2 and those in exon 3 were replaced with human ones). All plasmids were verified by complete sequencing.

### Viruses

The SARS-CoV-2 isolates were propagated in Vero E6-TMPRSS2 cells in the BSL-3 laboratory. Briefly, 3 × 10^6^ Vero E6 cells were plated into a T75 flask in DMEM supplemented with 2% FBS. After 24 h, cells were inoculated with 0.001 or 0.01 multiplicity of infection (MOI) of SARS-CoV-2 D614G (hCoV-19/Italy/LOM-UniMI-vir1/2020; EPI_ISL_58405), SARS-CoV-2 B.1.617.2 Delta (hCoV-19/Italy/LOM-Milan-UNIMI9615/2021, EPI_ISL_3073880), SARS-CoV-2 B.1.1.529 Omicron (hCoV-19/Italy/LOM-19182/2021, EPI_ISL_10898045) and mouse-adapted SARS-CoV-2 (rSARS2-N501Y_MA30_)^37^. The supernatant was collected 48–72 h later, centrifuged for 5 min at 500*g*, aliquoted and stored at −80 °C. Virus stocks were titrated by plaque assay. Briefly, Vero E6-TMPRSS2 cells were inoculated in replication medium (complete DMEM supplemented with 2% FBS) with serially diluted filtered supernatants collected from infected cells for 1 h, and the medium was replaced with overlay medium containing 0.6% agar in MEM. The cells were fixed by the addition of 4% paraformaldehyde 3 days postinfection and cell monolayers were stained for 15 min with crystal violet 1% dissolved in 10% ethanol for plaques counting and titer calculation in PFU per ml.

In Extended Data Fig. 2a, the sequences of SARS-CoV-2 variants (D614G, B.1.617.2, B.1.1.529) were aligned using Snapgene version 5.1.7.

### In vitro infection

3T3 cells expressing hACE2, mACE2 and hyACE2 were seeded in 96-well plates at 5,000 cells per well and infected 24 h later with 0.01, 0.1 and 1 MOI in complete medium with 2% FBS. After 48 and 72 h, infection was assessed by viral cytopathic effect and SARS-CoV-2 genome quantification. Cytopathic effect was assessed using the luminescent-bases CellTiter-Glo Assay (Promega) and Infinite F200 plate reader (Tecan). Relative luciferase units (RLUs) were normalized to infected or uninfected controls to obtain the percentage of inhibition of cytopathic effect using the following formula: % CPE inhibition = 100 × (test Cmpd − avg. virus)/(avg. cells − avg. virus), where Avg. virus is the RLU average obtained from infected and not treated wells and Avg. cells is the RLU average obtained from not infected and not treated wells. Dose–response curves were generated by nonlinear regression curve fitting with GraphPad Prism to calculate IC_50_.

For SARS-CoV-2 genome quantification, cell supernatants were subjected to direct lysis with the addition of 10 µl ViRNAex solution (Cabru) and heated at 70 °C for 15 min. After the addition of distilled water (1:2), samples were used as templates for PCR amplification using TaqPath 1-Step RT-qPCR Master Mix (Thermo Fisher Scientific) and specific SARS-CoV-2 primers/probe (2019-nCoV RUO, Integrated DNA Technologies) and standard (2019_nCOV_N positive control, Integrated DNA Technologies) to determine viral copy number. Obtained Ct were normalized to untreated infected wells, and dose–response curves were generated by nonlinear regression curve fitting with GraphPad Prism to calculate the concentration that inhibits 50% of viral replication (IC_50_).

### Mice

D_H_LMP2a mice^27^ were originally provided by K. Rajewsky (Harvard Medical School) and bred >10 generations against C57BL/6 mice. B6.Cg-Tg(K18-ACE2)^2Prlmn/^J mice^28^ (referred to in the text as K18-hACE2) were purchased from the Jackson Laboratory. C57BL/6 mice were purchased from Charles River. Male mice at 8–10 weeks of age were used for experiments.

hyACE2 knock-in mice were generated by nucleofecting male embryonic stem (ES) cells (hemizygote for mACE2, as the ACE2 gene is located on the X chromosome) obtained in-house from matings of C57BL/6N and 129S2/Sv mice. ES cells were nucleofected with Cas9 protein armed with two RNA guides that cut inside exon 2 and exon 3 of the mACE2 gene (exon 1 is untranslated) and a ‘megamer’ 13.5 kb donor DNA fragment. The megamer covers the most downstream part of mouse intron 1, exon 2 (the leader sequence is mouse, the rest of the exon is human), mouse intron 2, human exon 3 and the most upstream part of mouse intron 3. The megamer was obtained by annealing several partially overlapping DNA molecules (chemically synthesized by Genewiz) and filling in with Klenow polymerase. About 200 ES cell clones were screened for the junction of mouse intron 1 and human exon 2 and the junction of human exon 3 and mouse exon 3. Four clones were positive, and the PCR products covering the junctions were sequenced. The positive clones were injected in morulas of C57BL/6N mice; all gave rise to male chimeric mice that transmitted the *hyACE2* allele to progeny when mated to C57BL/6N females. Both hemizygous male and homozygous female mice bearing the *hyACE2* allele were viable and fertile, with no detectable difference from WT mice. The hyACE2 mice were backcrossed >10 times into the C57BL/6N background before using them in the experiments described here. hyACE2 homozygous females or hemizygous males were used at 8–10 weeks of age.

Mice had ad libitum access to drinking water and chow (VRF1 standard diet, Safe, 801900). Mice were housed under specific pathogen-free conditions with a 12-h light/12-h dark cycle, a temperature ranging between 20 °C and 23 °C and 60% humidity.

All experimental animal procedures were approved by the Institutional Animal Committee of the San Raffaele Scientific Institute (authorization 270/2022-PR), and all infectious work was performed in designated BSL-3 workspaces.

### SARS-CoV-2 infection

Infection of K18-hACE2 transgenic mice, hyACE2 knock-in mice and C57BL/6 mice with aerosolized SARS-CoV-2 was performed as described^29^. Briefly, nonanesthetized mice were placed in a nose-only Allay restrainer on the inhalation chamber (DSI Buxco respiratory solutions; DSI). To reach a target accumulated inhaled aerosol (also known as delivered dose) as indicated in the figure legends, mice were exposed to aerosolized SARS-CoV-2 D614G, B.1.617.2 Delta or B.1.1.529 Omicron for 40–60 min (depending on the total volume of diluted virus and on the number of mice simultaneously exposed). In selected experiments, C57BL/6 mice were exposed to a target accumulated inhaled aerosol of the mouse-adapted SARS-CoV-2 strain (rSARS-N501Y_MA30_). Primary inflows and pressure were controlled and set to 0.5 l min^−1^ per port and −0.5 cmH_2_O, respectively. As control, K18-hACE2 mice, hyACE2 knock-in mice and C57BL/6 mice received the same volume of aerosolized PBS (125 μl per mouse). Infected mice were monitored daily to record body weight and clinical and respiratory parameters.

The clinical score was based on a cumulative 0–3 scale evaluating fur, posture, activity level, eyes and breathing^29^.

### mRNA-LNP vaccine production

The codon-optimized sequences for S-2P (SARS-CoV-2 Wuhan full-length spike with K986P and V987P amino acid substitutions) and firefly luciferase (Luc) were synthesized by GenScript and cloned into an mRNA production plasmid as previously described^38^. The plasmids were linearized, and mRNAs were generated using MEGAscript T7 RNA polymerase (Ambion). mRNAs were transcribed to contain poly(A) tails of 101 nucleotides in length. Uridine 5′-triphosphates were substituted for N1-methylpseudouridine 5′-triphosphates (TriLink), and cap1 structure was generated using CleanCap (TriLink). mRNAs were purified by cellulose purification as previously described^52^ and analyzed by agarose gel electrophoresis. Purified mRNAs were encapsulated in LNPs using a self-assembly process by rapidly mixing an aqueous solution of mRNA at pH 4.0 with a solution of lipids dissolved in ethanol. LNPs were similar in composition to those described previously^53^, which contain an ionizable lipid/phosphatidylcholine/cholesterol/polyethylene glycol-lipid. The ionizable lipid is proprietary of Acuitas Therapeutics and described in US patent US10221127. The LNPs had a diameter of ~80 nm as measured by dynamic light scattering using a Zetasizer Nano ZS (Malvern Instruments) instrument.

### In vivo treatment

hyACE2 knock-in mice were vaccinated via intramuscular injection into the gastrocnemius muscle with 10 μg and 5 μg (28 days later) of SARS-CoV-2 full-length spike (S-2P) mRNA-LNP or luciferase mRNA-LNP^54^. After 28 days, mice received an immunization boost with 5 μg SARS-CoV-2 S-2P mRNA-LNP or luciferase mRNA-LNP. SARS-Cov-2 S-2P mRNA vaccines were designed based on the SARS-CoV-2 full-length spike (S) protein sequence with K986P and V987P amino acids substitutions (Wuhan-Hu-1, GenBank: MN908947.3)^38^.

For the experiments described in Fig. 6, mice were injected intravenously with 200 μg per mouse of anti-CD4 (clone GK1.5, BioXcell), anti-CD8 (clone YTS169.4, BioXcell) or both, three times 2 days apart. In addition, a group of mice was injected intravenously with 250 μg per mouse of anti-mouse IFN-γ (clone XMG1.2, BioXcell) two times, 4 h before and 3 days after the viral challenge.

In the experiments described in Fig. 4, mice were treated with 50 μg per mouse of Treg-Protector (anti-ARTC2 nanobody; clone: S + 16a; BioLegend, 149802) by intravenous injection 30 min before sacrificing them.

### WBP

WBP was performed using a WBP chamber (DSI Buxco respiratory solutions; DSI) as described in ref. ^29^. Mice were allowed to acclimate inside the chamber for 8 min before recording respiratory parameters for 15 min using FinePointe software.

### Viral titers

Lungs were perfused and homogenized in M tubes (Milteny BioTec, 130-093-335) containing 1 ml of DMEM supplemented with 0% FBS, using gentleMACS Octo Dissociator (130-096-427). Samples underwent three cycles with program m_Lung_01_02 (34 s, 164 rpm). After centrifugation at 2,328*g* for 5 min at 4 °C, supernatants were stored at −80 °C for viral isolation and viral load detection. Viral titer was determined by TCID_50_. Vero E6 cells were seeded in flat-bottom 96-well tissue culture plates (1.5 × 10^4^ cells per well). The day after, 10-fold dilutions of the homogenized tissue were applied to confluent cells and incubated for 1 h at 37 °C. Cells were washed with PBS and incubated for 72 h at 37 °C in DMEM supplemented with 2% FBS. After fixation with 4% paraformaldehyde for 30 min, cells were stained with 0.05% (wt/vol) crystal violet in 20% ethanol. The limit of detection (LOD) was defined as the lowest concentration whereby the virus, used as a positive control, has a killing capacity of cells.

### RNA extraction and qPCR

Tissues homogenates were prepared by homogenizing perfused lung, NTs, stomach, kidney, liver, heart, olfactory bulbs (OBs) and brain using gentleMACS dissociator (Miltenyi Biotec, 130-096-427) with program RNA_02 in M tubes (130-096-335) in 500 μl (NTs and OBs) or 1 ml of Trizol (Invitrogen, 15596018). The program was run twice for selected organs (stomach, kidney and small intestine). The homogenates were centrifuged at 2,000*g* for 1 min at 4 °C, and the supernatant was collected. RNA extraction was performed by combining phenol/guanidine-based lysis with silica membrane-based purification. Briefly, 100 μl of chloroform was added to 500 μl of homogenized sample and total RNA was extracted using ReliaPrep RNA Tissue Miniprep column (Promega, Z6111). Total RNA was isolated according to the manufacturer’s instructions. qPCR was performed using TaqMan Fast virus 1-Step PCR Master Mix (Life Technologies, 4444434). Standard curve was drawn with 2019_nCOV_N positive control (IDT, 10006625). Primers used are as follows: 2019-nCoV_N1-forward primer (5′-GAC CCC AAA ATC AGC GAA AT-3′), 2019-nCoV_N1-reverse primer (5′-TCT GGT TAC TGC CAG TTG AAT CTG-3′) and 2019-nCoV_N1-probe (5′-FAM-ACC CCG CAT TAC GTT TGG TGG ACC-BHQ1-3′; Centers for Disease Control and Prevention). In Extended Data Fig. 2e, human or mouse ACE2 expression was analyzed on total RNA extracted as above. Genomic DNA was digested with a Turbo DNA-free TM kit (Life Technologies, AM1907), RNA was retro-transcribed to cDNA with Superscript IV Vilo (Life Technologies, 11756050), qPCR was performed in TaqMan Fast Universal PCR Master Mix (Life Technologies, 4364103). Primers used allow the amplification of exons 2 and 3 of ACE2 (hyACE2 forward: TAACCACGAAGCCGAAGAC, hyACE2 reverse: TCTGAGAGCACTGAAGACC; mACE2 forward: TTGTTGCTGTTACTACTGCTC, mACE2 reverse: CTGAAGACCCACTTTGCTG). The probes used are as follows: hyACE2 probe (AAAGGAACAGTCCACACTTGCCCAAATG); mACE2 probe (AGAAATCCAGACTCCGATCATCAAGCGTCA). All experiments were performed in duplicate.

### ELISA

Individual sera were titrated in parallel for the presence of SARS-CoV-2 S1 RBD-specific antibody by end-point ELISA. The ELISA plates were functionalized by coating with recombinant SARS-CoV-2 S1 subunit protein (RayBiotech, 230-30162) at a concentration of 2 μg ml^−1^ and incubated overnight (O/N) at 4 °C. Subsequently, the plates were blocked with 3% fat-free milk, 0.05% Tween 20 in PBS for 1 h at room temperature. The sera were then added at a dilution of 1/20 (sera from day 7) or 1/500 (sera from days 14, 21 and 28) and diluted 1:10 up to 1/1,280 or 1/32,000, respectively, in duplicate, and the plates were incubated for 2 h at room temperature. After five washes with 0.05% Tween 20 in PBS, the secondary anti-mouse IgG conjugated with horseradish peroxidase (PerkinElmer, NEF822001EA; 1:2,000) was added and the plates were incubated for 1 h at room temperature. After washing, the binding of the secondary antibody was detected by adding the substrate 3,3′,5,5′-tetramethylbenzidine (BD Biosciences). The reaction was blocked with 0.5 M H_2_SO_4_ and the absorbance at 450 nm and reference 630 nm was measured.

### Cell isolation and flow cytometry

Mice were killed by cervical dislocation. At the time of autopsy, mice were perfused through the right ventricle with PBS. Brain and OBs were removed from the skull and NTs from the nasal cavity. Lung tissue was digested in Roswell Park Memorial Institute 1640 containing 3.2 mg ml^−1^ Collagenase IV (Sigma, C5138) and 25 U ml^−1^ DNAse I (Sigma, D4263) for 30 min at 37 °C. Homogenized lungs were passed through 70 μm nylon meshes to obtain a single-cell suspension. Cells were resuspended in 36% Percoll solution (Sigma, P4937) and centrifuged for 20 min at 760*g* (light acceleration and low brake). The remaining red blood cells were removed with ammonium–chloride–potassium (ACK) lysis. Peripheral blood was collected in PBS 0.5 mM EDTA and lysed two times with ACK. In selected experiments, spleen and mediastinal lymph nodes were collected before lung perfusion. Single-cell suspensions were generated as described^55^.

For analysis of ex vivo intracellular cytokine production, 1 mg ml^−1^ of brefeldin A (Sigma, B7651) was included in the digestion buffer. All flow cytometry stainings of surface-expressed and intracellular molecules were performed as described in refs. ^29,56–58^. Briefly, cells were stimulated for 4 h at 37 °C in the presence of brefeldin A, monensin (Life Technologies, 00-4505-51) and a pool of overlapping peptides (1 μg ml^−1^ per peptide), including MHC class I- and MHC class II-restricted peptides (9–22 amino acids) covering the S, S1, S+, M and N proteins of SARS-CoV-2 (Miltenyi Biotec, 130-126-700, 130-127-041, 130-127-311, 130-126-702 and 130-126-698)^31^. As a positive control for IFN-γ and TNF production, cells were stimulated with PMA (Invitrogen, 356150050) and ionomycin (Invitrogen, I24222). Cell viability was assessed by staining with Viobility 405/520 fixable dye (Miltenyi Biotec, 130-109-814). In Fig. 1n–q and Extended Data Fig. 1e, biotinylated-RDB (26 KDa, kindly provided by G. Roscilli and L. Aurisicchio; Takis Biotech) was mixed with Alexa Fluor (AF)-647 or 488-conjugated streptavidin (53 kDa) at a molar ratio of 4:1. RBD-specific B cells were identified by labeling them, before surface staining, with 2 μg ml^−1^ of RBD-tetramers for 30 min at 4 °C. Antibodies (Abs) used for flow cytometry stainings are indicated in Supplementary Table 1.

Flow cytometry analysis was performed on BD FACS Symphony A5 SORP using BD FACS Diva; or Cytek Aurora (five laser configuration) using SpectroFlow 3.2.0. Data were analyzed with FlowJo software 10.5.3 (Treestar).

### Histochemistry

Mice were killed and perfused transcardially with PBS. One left lobe of the lung was fixed in zinc formalin and transferred into 70% ethanol 24 h later. Tissues were then processed, embedded in paraffin and automatically stained for SARS-CoV-2 nucleocapsid (Sino Biological, 40143-R019) through LEICA BOND RX for 1 h at room temperature and developed with Bond Polymer Refine Detection (Leica Biosystems, DS9800). Bright-field images were acquired with an Aperio Scanscope System CS2 microscope and the ImageScope program (Leica Biosystems) following the manufacturer’s instructions.

### Confocal immunofluorescence histology

After killing and transcardial perfusion with PBS, the left lung lobe of the mouse was recovered, fixed in 4% paraformaldehyde for 16 h, dehydrated in 30% sucrose and embedded in optimal cutting temperature freezing media (Killik Bio-Optica, 05-9801). Sections (20 μm) were cut on a CM1520 cryostat (Leica Biosystems), adhered to Superfrost Plus slides (Thermo Fisher Scientific), permeabilized and blocked in PBS containing 0.3% Triton X-100 (Sigma-Aldrich) and 0.5% BSA. Staining was performed in PBS containing 0.1% Triton X-100 and 0.2% BSA. Slides were stained for T cell receptor-β (TCR-β; clone H57-597; BioLegend, 109218) and B220 (clone RA3-6B2; BioLegend, 103228) overnight at 4 °C. Sections were washed twice for 5 min and stained with DAPI (Life Technologies, D1360) for 5 min at room temperature. After a final washing step, sections were mounted with FluorSaveTM Reagent (Merck Millipore, 345789) for imaging on SP5 or SP8 confocal microscopes with 40× objectives (Leica Microsystems). The Leica sequential laser excitation and detection modality were used to minimize spectral spillover.

### Statistical analyses and software

Detailed information concerning the statistical methods used is provided in the last sentence of all figure legends. Flow data were collected using FlowJo Version 10.5.3 (Treestar). Statistical analyses were performed with GraphPad Prism software version 8 (GraphPad). Immunohistochemical imaging analyses were performed with QuPath 0.2.3 (Quantitative Pathology & Bioimage 5 Analysis) software. Data collection and analysis were not performed blind to the conditions of the experiments. *n* represents individual mice analyzed per experiment. No statistical methods were used to predetermine sample sizes but our sample sizes are similar to those reported in previous publications^29,59^. Age-matched and sex-matched animals were randomly assigned to each group. Experiments were performed independently at least twice to control for experimental variation. Error bars indicate the standard error of the mean (s.e.m.). We used Mann–Whitney *U* tests to compare two groups with nonnormally distributed continuous variables and Kruskal–Wallis nonparametric test or one-way analysis of variance (ANOVA) test to compare three or more unpaired groups. Normality of data distribution was tested with a Shapiro–Wilk normality test, and normality was chosen only when normality could be confirmed for each dataset. We used two-way ANOVA followed by Fisher’s LSD test to analyze experiments with multiple groups, two independent variables and each condition stands alone. Kaplan–Meier curves were compared with the log-rank (Mantel–Cox) test. Significance is indicated as follows: *P < 0.05; **P < 0.01; ***P < 0.001. Comparisons are not statistically significant unless indicated.

### Reporting summary

Further information on research design is available in the Nature Portfolio Reporting Summary linked to this article.

## Online content

Any methods, additional references, Nature Portfolio reporting summaries, source data, extended data, supplementary information, acknowledgements, peer review information; details of author contributions and competing interests; and statements of data and code availability are available at 10.1038/s41590-024-01787-z.

### Supplementary information

Supplementary Information Supplementary Table 1.

Reporting Summary

### Source data

Source Data Fig. 1 Statistical source data.

Source Data Fig. 2 Statistical source data.

Source Data Fig. 3 Statistical source data.

Source Data Fig. 4 Statistical source data.

Source Data Fig. 5 Statistical source data.

Source Data Fig. 6 Statistical source data.

Source Data Extended Data Fig. 1 Statistical source data.

Source Data Extended Data Fig. 2 Statistical source data.

Source Data Extended Data Fig. 3 Statistical source data.

Source Data Extended Data Fig. 4 Statistical source data.

Source Data Extended Data Fig. 5 Statistical source data.

Source Data Extended Data Fig. 6 Statistical source data.

Source Data Extended Data Fig. 7 Statistical source data.

## Data Availability

All data are available in the paper and supplementary files or from the corresponding authors upon reasonable request. The electron microscopy structure of the ACE2/RBD complex is available in the PDB under the code 6M17. Source data are provided with this paper.

## References

[1] Khoury DS (2021). Neutralizing antibody levels are highly predictive of immune protection from symptomatic SARS-CoV-2 infection. Nat. Med..

[2] Cromer D (2023). Predicting vaccine effectiveness against severe COVID-19 over time and against variants: a meta-analysis. Nat. Commun..

[3] Schäfer A (2020). Antibody potency, effector function, and combinations in protection and therapy for SARS-CoV-2 infection in vivo. J. Exp. Med..

[4] Tseng HF (2022). Effectiveness of mRNA-1273 against SARS-CoV-2 Omicron and Delta variants. Nat. Med..

[5] Sievers BL (2022). Antibodies elicited by SARS-CoV-2 infection or mRNA vaccines have reduced neutralizing activity against Beta and Omicron pseudoviruses. Sci. Transl. Med..

[6] Seow J (2020). Longitudinal observation and decline of neutralizing antibody responses in the three months following SARS-CoV-2 infection in humans. Nat. Microbiol..

[7] Chia WN (2021). Dynamics of SARS-CoV-2 neutralising antibody responses and duration of immunity: a longitudinal study. Lancet Microbe.

[8] Robbiani DF (2020). Convergent antibody responses to SARS-CoV-2 in convalescent individuals. Nature.

[9] Iketani S (2022). Antibody evasion properties of SARS-CoV-2 Omicron sublineages. Nature.

[10] Case JB (2022). Resilience of S309 and AZD7442 monoclonal antibody treatments against infection by SARS-CoV-2 Omicron lineage strains. Nat. Commun..

[11] McCallum M (2022). Structural basis of SARS-CoV-2 Omicron immune evasion and receptor engagement. Science.

[12] Sette A, Sidney J, Crotty S (2023). T cell responses to SARS-CoV-2. Annu. Rev. Immunol..

[13] Israelow B (2021). Adaptive immune determinants of viral clearance and protection in mouse models of SARS-CoV-2. Sci. Immunol..

[14] Quinti I (2020). A possible role for B cells in COVID-19? Lesson from patients with agammaglobulinemia. J. Allergy Clin. Immunol..

[15] Soresina A (2020). Two X‐linked agammaglobulinemia patients develop pneumonia as COVID‐19 manifestation but recover. Pediatr. Allergy Immunol..

[16] Cohen B (2021). COVID-19 infection in 10 common variable immunodeficiency patients in New York City. J. Allergy Clin. Immunol. Pract..

[17] Bange EM (2021). CD8^+^ T cells contribute to survival in patients with COVID-19 and hematologic cancer. Nat. Med..

[18] Apostolidis SA (2021). Cellular and humoral immune responses following SARS-CoV-2 mRNA vaccination in patients with multiple sclerosis on anti-CD20 therapy. Nat. Med..

[19] Brill L (2021). Humoral and T-cell response to SARS-CoV-2 vaccination in patients with multiple sclerosis treated with ocrelizumab. JAMA Neurol..

[20] Iannetta M (2021). T-cell responses to SARS-CoV-2 in multiple sclerosis patients treated with ocrelizumab healed from COVID-19 with absent or low anti-spike antibody titers. Mult. Scler. Relat. Disord..

[21] Endres R (1999). Mature follicular dendritic cell networks depend on expression of lymphotoxin β receptor by radioresistant stromal cells and of lymphotoxin β and tumor necrosis factor by B cells. J. Exp. Med..

[22] Nolte MA (2004). B cells are crucial for both development and maintenance of the splenic marginal zone. J. Immunol..

[23] Tumanov AV (2002). Distinct role of surface lymphotoxin expressed by b cells in the organization of secondary lymphoid tissues. Immunity.

[24] Phan TG, Green JA, Gray EE, Xu Y, Cyster JG (2009). Immune complex relay by subcapsular sinus macrophages and noncognate B cells drives antibody affinity maturation. Nat. Immunol..

[25] Moseman EA (2012). B cell maintenance of subcapsular sinus macrophages protects against a fatal viral infection independent of adaptive immunity. Immunity.

[26] Homann D (1998). Evidence for an underlying CD4 helper and CD8 T-cell defect in B-cell-deficient mice: failure to clear persistent virus infection after adoptive immunotherapy with virus-specific memory cells from μMT/μMT mice. J. Virol..

[27] Casola S (2004). B cell receptor signal strength determines B cell fate. Nat. Immunol..

[28] McCray PB (2007). Lethal infection of K18-hACE2 mice infected with severe acute respiratory syndrome coronavirus. J. Virol..

[29] Fumagalli V (2021). Administration of aerosolized SARS-CoV-2 to K18-hACE2 mice uncouples respiratory infection from fatal neuroinvasion. Sci. Immunol..

[30] Wellford SA (2022). Mucosal plasma cells are required to protect the upper airway and brain from infection. Immunity.

[31] Silva-Cayetano A (2021). A booster dose enhances immunogenicity of the COVID-19 vaccine candidate ChAdOx1 nCoV-19 in aged mice. Med.

[32] Yan R (2020). Structural basis for the recognition of SARS-CoV-2 by full-length human ACE2. Science.

[33] Wang Q (2020). Structural and functional basis of SARS-CoV-2 entry by using human ACE2. Cell.

[34] Ren W (2021). Comparative analysis reveals the species-specific genetic determinants of ACE2 required for SARS-CoV-2 entry. PLoS Pathog..

[35] Shang J (2020). Structural basis of receptor recognition by SARS-CoV-2. Nature.

[36] Li F, Li W, Farzan M, Harrison SC (2005). Structure of SARS coronavirus spike receptor-binding domain complexed with receptor. Science.

[37] Wong L-YR (2022). Eicosanoid signaling blockade protects middle-aged mice from severe COVID-19. Nature.

[38] Laczkó D (2020). A single immunization with nucleoside-modified mRNA vaccines elicits strong cellular and humoral immune responses against SARS-CoV-2 in mice. Immunity.

[39] Guidotti LG, Chisari FV (2001). Noncytolytic control of viral infections by the innate and adaptive immune response. Annu. Rev. Immunol..

[40] Zhao J (2016). Airway memory CD4^+^ T cells mediate protective immunity against emerging respiratory coronaviruses. Immunity.

[41] Sun S-H (2020). A mouse model of SARS-CoV-2 infection and pathogenesis. Cell Host Microbe.

[42] Zhou B (2021). SARS-CoV-2 spike D614G change enhances replication and transmission. Nature.

[43] Zhou X (2023). A novel hACE2 knock-in mouse model recapitulates pulmonary and intestinal SARS-CoV-2 infection. Front. Microbiol..

[44] Nakandakari-Higa S (2022). A minimally-edited mouse model for infection with multiple SARS-CoV-2 strains. Front. Immunol..

[45] Bert NL (2020). SARS-CoV-2-specific T cell immunity in cases of COVID-19 and SARS, and uninfected controls. Nature.

[46] Tarke A (2022). SARS-CoV-2 vaccination induces immunological T cell memory able to cross-recognize variants from Alpha to Omicron. Cell.

[47] Geers D (2021). SARS-CoV-2 variants of concern partially escape humoral but not T cell responses in COVID-19 convalescent donors and vaccine recipients. Sci. Immunol..

[48] Bertoletti A, Bert NL, Tan AT (2022). SARS-CoV-2-specific T cells in the changing landscape of the COVID-19 pandemic. Immunity.

[49] Zonozi R (2023). T cell responses to SARS-CoV-2 infection and vaccination are elevated in B cell deficiency and reduce risk of severe COVID-19. Sci. Transl. Med..

[50] Bertoletti A, Bert NL, Qui M, Tan AT (2021). SARS-CoV-2-specific T cells in infection and vaccination. Cell. Mol. Immunol..

[51] Maier JA (2015). ff14SB: improving the accuracy of protein side chain and backbone parameters from ff99SB. J. Chem. Theory Comput..

[52] Baiersdörfer M (2019). A facile method for the removal of dsRNA contaminant from in vitro-transcribed mRNA. Mol. Ther. Nucleic Acids.

[53] Maier MA (2013). Biodegradable lipids enabling rapidly eliminated lipid nanoparticles for systemic delivery of RNAi therapeutics. Mol. Ther..

[54] Alameh M-G (2021). Lipid nanoparticles enhance the efficacy of mRNA and protein subunit vaccines by inducing robust T follicular helper cell and humoral responses. Immunity.

[55] Sammicheli S (2016). Inflammatory monocytes hinder antiviral B cell responses. Sci. Immunol..

[56] Bénéchet AP (2019). Dynamics and genomic landscape of CD8^+^ T cells undergoing hepatic priming. Nature.

[57] Fumagalli V (2022). Group 1 ILCs regulate T cell-mediated liver immunopathology by controlling local IL-2 availability. Sci. Immunol..

[58] Simone GD (2021). Identification of a Kupffer cell subset capable of reverting the T cell dysfunction induced by hepatocellular priming. Immunity.

[59] Fumagalli V (2023). Nirmatrelvir treatment of SARS‐CoV‐2‐infected mice blunts antiviral adaptive immune responses. EMBO Mol. Med..

