# Supplementary material for: Antibody-independent protection against heterologous SARS-CoV-2 challenge conferred by prior infection or vaccination
Source: Nat Immunol. 2024 Mar 14;25(4):633–43. doi: 10.1038/s41590-024-01787-z (PMC11003867; doi:10.1038/s41590-024-01787-z)
Supplement: Supplementary file 1 — Supplementary Table 1. [file 41590_2024_1787_MOESM1_ESM.pdf]

nature immunology

Article

https://doi.org/10.1038/s41590-024-01787-z

Antibody-independent protection against heterologous SARS-CoV-2 challenge conferred by prior infection or vaccination

In the format provided by the authors and unedited

Table 1. List of antibodies used for flow cytometry

Name	Clone	Source and catalog number	Dilution
CD8	53-6.7	BD Biosciences #558106, Biolegend #100759; BD Horizon #566096	1:100
CD4	RM4-5<br><br>GK1.5	Biolegend #100548; BD Biosciences #740208<br>BD Pharmingen #568695	1:100
B220	RA3-6B2	BD Biosciences #564662	1:100
CD19	1D3	BD Biosciences #749027	1:100
CD44	IM7	BD Biosciences #741227; BioLegend #103028	1:150
CD69	H1.2F3	BD Biosciences #612793; Biolegend#104537	1:100
CD25	PC61	BD Bioscences #564023	1:100
CD19	1D3	BD Biosciences #749027	1:100
CD62L	MEL-4	Biolegend #104453; Biolegend #161205	1:100
CD45	30-F11	Biolegend #103113, BD Biosciences #564279	1:100
IFN-γ	XMG1.2	BD Biosciences #557735	1:100
TNF	MP6-XT22	Biolegend #506329	1:100
CD279 (PD-1)	RMP1-30	BD Biosciences #749306; Biolegend #135257	1:100
Granzyme-B	GB12	Invitrogen #MHGB04	1:80
CD86	GL1	BD Biosciences #564199	1:100
CD80	16-10A1	Biolegend #104738	1:100
CD95 (FAS)	Jo2	BD Biosciences #557653	1:100
GL7	GL7	Biolegend # 144612	1:80
Bcl6	K112-91	BD Biosciences #562401	1:80
CXCR5	2G8	Biolegend #145532	1:80
T-bet	4B10	Invitrogen #25-5825-80; Biolegend #644805	1:100
ICOS (CD278)	C398.4A	Biolegend #313537	1:100
CD138	231-2	Biolegend 142510	1:100
CXCR3 (CD183)	CXCR3-173	BD Bioscences #741895; Biolegend #126505	1:100
CD11a	2D7	Biolegend #101005	1:100
CD49d	R1-2	Biolegend #103625	1:100
CD103	2E7	Biolegend #121407	1:100
Streptavidin-AF647		Invitrogen # S32357	
Streptavidin-AF488		Invitrogen #S32354	
