# Supplementary material for: Antibody-independent protection against heterologous SARS-CoV-2 challenge conferred by prior infection or vaccination
Source: Nat Immunol. 2024 Mar 14;25(4):633–43. doi: 10.1038/s41590-024-01787-z (PMC11003867; doi:10.1038/s41590-024-01787-z)
Supplement: Supplementary file 2 — Reporting Summary [file 41590_2024_1787_MOESM2_ESM.pdf]

nature portfolio

Corresponding author(s): Marco E.Bianchi
Luca G. Guidotti
Matteo Iannacone

Last updated by author(s): Sep 29, 2023

Reporting Summary

Nature Portfolio wishes to improve the reproducibility of the work that we publish. This form provides structure for consistency and transparency in reporting. For further information on Nature Portfolio policies, see our Editorial Policies and the Editorial Policy Checklist.

Statistics

For all statistical analyses, confirm that the following items are present in the figure legend, table legend, main text, or Methods section.

n/a	Confirmed	
☐	☒	The exact sample size (*n*) for each experimental group/condition, given as a discrete number and unit of measurement
☐	☒	A statement on whether measurements were taken from distinct samples or whether the same sample was measured repeatedly
☐	☒	The statistical test(s) used AND whether they are one- or two-sided<br>*Only common tests should be described solely by name; describe more complex techniques in the Methods section.*
☒	☐	A description of all covariates tested
☐	☒	A description of any assumptions or corrections, such as tests of normality and adjustment for multiple comparisons
☐	☒	A full description of the statistical parameters including central tendency (e.g. means) or other basic estimates (e.g. regression coefficient) AND variation (e.g. standard deviation) or associated estimates of uncertainty (e.g. confidence intervals)
☐	☒	For null hypothesis testing, the test statistic (e.g. F, t, r) with confidence intervals, effect sizes, degrees of freedom and P value noted<br>*Give P values as exact values whenever suitable.*
☒	☐	For Bayesian analysis, information on the choice of priors and Markov chain Monte Carlo settings
☒	☐	For hierarchical and complex designs, identification of the appropriate level for tests and full reporting of outcomes
☒	☐	Estimates of effect sizes (e.g. Cohen's *d*, Pearson's *r*), indicating how they were calculated

Our web collection on statistics for biologists contains articles on many of the points above.

Software and code

Policy information about availability of computer code

Data collection	for in silico mutation: "Mutate residue" tool available in Maestro 9.9 (Schrodinger Suite)<br>for flow-cytometry: BD FACS Diva (Symphony A5 SORP) or SpectroFlow3.2.0 (Cytek Aurora (5 laser configuration))<br>for imaging: SP5 or SP8 confocal microscopes with 40x objectives (Leica Microsystem); ImageScope program (Leica Biosystem)<br>for respiratory parameters: FinePoint Software
Data analysis	for sequence alignment: NCBI BLAST+, EMBOSS NEeedle online tools<br>for flow cytometry: FlowJo software 10.5.3<br>for raw data analyses: GraphPad Prism 8<br>for imaging: QuPath 0.2.3

For manuscripts utilizing custom algorithms or software that are central to the research but not yet described in published literature, software must be made available to editors and reviewers. We strongly encourage code deposition in a community repository (e.g. GitHub). See the Nature Portfolio guidelines for submitting code & software for further information.

Data

Policy information about availability of data

All manuscripts must include a data availability statement. This statement should provide the following information, where applicable:

- Accession codes, unique identifiers, or web links for publicly available datasets
- A description of any restrictions on data availability
- For clinical datasets or third party data, please ensure that the statement adheres to our policy

All data are available in the main text or in the extended data. Source data are provided with this paper. Electron microscopy structure of ACE2/RBD complex: PDB code 6M17
(DOI: https://doi.org/10.2210/pdb6M17/pdb).

Research involving human participants, their data, or biological material

Policy information about studies with human participants or human data. See also policy information about sex, gender (identity/presentation), and sexual orientation and race, ethnicity and racism.

Reporting on sex and gender	N/A
Reporting on race, ethnicity, or other socially relevant groupings	N/A
Population characteristics	N/A
Recruitment	N/A
Ethics oversight	N/A

Note that full information on the approval of the study protocol must also be provided in the manuscript.

Field-specific reporting

Please select the one below that is the best fit for your research. If you are not sure, read the appropriate sections before making your selection.

☒ Life sciences ☐ Behavioural & social sciences ☐ Ecological, evolutionary & environmental sciences

For a reference copy of the document with all sections, see nature.com/documents/nr-reporting-summary-flat.pdf

Life sciences study design

All studies must disclose on these points even when the disclosure is negative.

Sample size	Sample sizes were chosen based on prior research conducted in our laboratories to provide sufficient numbers of mice in each group to provide informative results and perform statistical testing. All experiments were highly reproducible. No statistical methods were used to predetermine sample sizes but our sample sizes are similar to those reported in previous publications (REF. PMID: 34812647; PMID: 36946379). For each independent experiment, minimum 3 mice per group were used.
Data exclusions	No data were excluded from analysis
Replication	Biological replicates were used to ensure reproducibility of this study. All presented data are representative of at least 2 independent experiments with similar results. All result described in the study could be reproduced.
Randomization	Mice were matched for age (8-10 weeks old) and sex before randomization. For in vitro experiments (Fig.2 and Extended Fig.2), cell line was used and experiments were performed in triplicate.
Blinding	Blinding was not performed as not relevant in this study, because subjective measurement was not involved.

Reporting for specific materials, systems and methods

We require information from authors about some types of materials, experimental systems and methods used in many studies. Here, indicate whether each material, system or method listed is relevant to your study. If you are not sure if a list item applies to your research, read the appropriate section before selecting a response.

Materials & experimental systems

n/a	Involved in the study
☐	☒ Antibodies
☐	☒ Eukaryotic cell lines
☒	☐ Palaeontology and archaeology
☐	☒ Animals and other organisms
☒	☐ Clinical data
☒	☐ Dual use research of concern
☒	☐ Plants

Methods

n/a	Involved in the study
☒	☐ ChIP-seq
☐	☒ Flow cytometry
☒	☐ MRI-based neuroimaging

Antibodies

Antibodies used

CD8 (clone 53-6.7 BD Biosciences #558106, Biolegend #100759; BD Horizon #566096)
CD4 (clone RM4-5 and GK1.5; Biolegend #100548; BD Biosciences #740208 BD Pharmingen #568695)
B220 (clone RA3-6B2 BD Biosciences #564662)
CD19 (clone 1D3 BD Biosciences #749027)
CD44 (clone IM7 BD Biosciences #741227; BioLegend #103028)
CD69 (clone H1.2F3 BD Biosciences #612793; Biolegend#104537)
CD25 (clone PC61 BD Bioscences #564023)
CD19 (clone 1D3 BD Biosciences #749027)
CD62L (clone MEL-4 Biolegend #104453; Biolegend #161205)
CD45 (clone 30-F11 Biolegend #103113, BD Biosciences #564279)
IFN-g (clone XMG1.2 BD Biosciences #557735)
TNF-α (clone MP6-XT22 Biolegend #506329)
CD279 (PD-1) (clone RMP1-30 BD Biosciences #749306; Biolegend #135257)
Granzyme-B (clone GB12 Invitrogen #MHGB04)
CD86 (clone GL1 BD Biosciences #564199)
CD80 (clone 16-10A1 Biolegend #104738)
CD95 (FAS) (clone Jo2 BD Biosciences #557653)
GL7 (clone GL7 Biolegend # 144612)
Bcl6 (clone K112-91 BD Biosciences #562401)
CXCR5 (clone 2G8 Biolegend #145532)
T-bet (clone 4B10 Invitrogen #25-5825-80; Biolegend #644805)
ICOS (CD278) (clone C398.4A Biolegend #313537)
CD138 (clone 231-2 Biolegend 142510)
CXCR3 (CD183) (clone CXCR3-173 BD Bioscences #741895; Biolegend #126505)
CD11a (clone 2D7 Biolegend #101005)
CD49d (clone R1-2 Biolegend #103625)
CD103 (clone 2E7 Biolegend #121407)
TCR-b (Biolegend, Clone H57-597, #109218)
Streptavidin-AF647 (Invitrogen # S32357)
Streptavidin-AF488 (Invitrogen #S32354)
anti-murine IgG conjugated with horseradish peroxidase (HRP, PerkinElmer, #NEF822001EA)

Validation

Antibodies with expression on leukocytes isolated from the lung of infected mice (e.g.CD8, CD4, B220, CD19, CD44, CD62L, IFN-g, TNF-a, FAS, etc.) and flurescently-labeled streptavidins have been previously used, validated, and published (see for instance Fumagalli et al., EMBO Mol Medicine 2023).

Eukaryotic cell lines

Policy information about cell lines and Sex and Gender in Research

Cell line source(s)

3T3 (ATCC CRL-1658)
Vero-E6 (ATCC CRL-1587)
Vero E6-TMPRSS2 (NIBSC 100978)

Authentication

Cell lines were not authenticated.

Mycoplasma contamination

Cell lines are routinely tested for mycoplasma contamination before use and tested negative for mycoplasma contamination (MycoAlert™ Mycoplasma Detection Kit Lonza, #LT07-118).

Commonly misidentified lines
(See ICLAC register)

Not listed in ICLAC

Animals and other research organisms

Policy information about studies involving animals; ARRIVE guidelines recommended for reporting animal research, and Sex and Gender in Research

Laboratory animals	DHLMP2A mice were originally provided by K. Rajewsky (Harvard Medical School) and bred >10 generations against C57BL/6 mice. B6.Cg-Tg(K18-ACE2)2Prlmn/J mice41 (referred to in the text as K18-hACE2) were purchased from The Jackson Laboratory. C57BL/6 mice were purchased from Charles River. Male mice at 8-10 weeks of age were used for experiments.<br>hyACE2 knock-in mice were generated by nucleofecting male ES cells (hemizygote for mACE2, as the ACE2 gene is located on the X chromosome) obtained in-house from matings of C57BL/6N and 129S2/Sv mice. ES cells were nucleofected with Cas9 protein armed with two RNA guides that cut inside exon 2 and exon 3 of the mACE2 gene (exon 1 is untranslated) and a “megamer” 13.5 kb donor DNA fragment. The megamer covers the most downstream part of mouse intron 1, exon 2 (the leader sequence is mouse, the rest of the exon is human), mouse intron 2, human exon 3 and the most upstream part of mouse intron 3. The megamer was obtained by annealing several partially overlapping DNA molecules (chemically synthesized by Genewiz) and filling in with Klenow polymerase. About 200 ES cell clones were screened for the junction of mouse intron 1 and human exon 2 and the junction of human exon 3 and mouse exon 3. Four clones were positive and the PCR products covering the junctions were sequenced. The positive clones were injected in morulas of C57BL/6N mice; all gave rise to male chimeric mice that transmitted the hyACE2 allele to progeny when mated to C57BL/6N females. Both hemizygous male and homozygous female mice bearing the hyACE2 allele were viable and fertile, with no detectable difference from wildtype mice. The hyACE2 mice were backcrossed >10 times into the C57BL/6N background before using them in the experiments described here. hyACE2 homozygous females or hemizygous males were used at 8-10 weeks of age.<br>Mice had ad libitum access to drinking water and chow (VRF1 standard diet, Safe, #801900). Mice were housed under specific pathogen-free conditions with a 12 hour light / 12 hour dark cycle, a temperature ranging between 20-23°C and 60% humidity.
Wild animals	No wild animals were used in the study
Reporting on sex	Male and Female mice were used in this study.
Field-collected samples	No field collected samples were included in the study
Ethics oversight	All experimental animal procedures were approved by the Institutional Animal Committee of the San Raffaele Scientific Institute and all infectious work was performed in designated BSL-3 workspaces.

Note that full information on the approval of the study protocol must also be provided in the manuscript.

Flow Cytometry

Plots

Confirm that:

☒ The axis labels state the marker and fluorochrome used (e.g. CD4-FITC).

☒ The axis scales are clearly visible. Include numbers along axes only for bottom left plot of group (a 'group' is an analysis of identical markers).

☒ All plots are contour plots with outliers or pseudocolor plots.

☒ A numerical value for number of cells or percentage (with statistics) is provided.

Methodology

Sample preparation	Sample preparation is described in the Materials & Methods section.
Instrument	BD FACS Symphony A5 SORP or CYtek Aurora
Software	BD FACS DIVA or SPectroFlow for acquisition and FlowJo software 10.5.3 for analyses
Cell population abundance	No sorted cells in this study.
Gating strategy	Gating strategies is indicated in the Figure legends and the Materials & Methods section. FIrstly, cells were gated to exclude doublets using side scatter high vs ares, then using forward and side scatter to identify events corresponding to lymphocytes. Living cells were selected by negativity for the viability dye (L/D NIR Fixable Viability dye). Afterwards B cells or T cells were gated based on the analyses required.

☐ Tick this box to confirm that a figure exemplifying the gating strategy is provided in the Supplementary Information.
